# Supplementary material for: Qualitative systematic review of general practitioners’ (GPs’) views and experiences of providing postnatal care
Source: BMJ Open. 2023 Apr 11;13(4):e070005. doi: 10.1136/bmjopen-2022-070005 (PMC10106050; doi:10.1136/bmjopen-2022-070005)
Supplement: Supplementary data [file bmjopen-2022-070005supp001.pdf]

Supplementary File 1: Search Terms

**MEDLINE**	Ovid MEDLINE(R) and In-Process, In-Data-Review & Other Non-Indexed Citations <1946 to September 27, 2021><br><br>1 General Practitioners/ 8852<br>2 Primary Health Care/ 83832<br>3 Physicians, Primary Care/ 3919<br>4 Physicians, Family/ 16693<br>5 (general practitioner* or GP*).mp. [mp=title, abstract, original title, name of substance word, subject heading word, floating sub-heading word, keyword heading word, organism supplementary concept word, protocol supplementary concept word, rare disease supplementary concept word, unique identifier, synonyms] 252660<br>6 1 or 2 or 3 or 4 or 5 340751<br>7 Perinatal Care/ 5026<br>8 Postpartum Period/ 27536<br>9 Postnatal Care/ 6048<br>10 Maternal Health/ 1909<br>11 exp Puerperal Disorders/ 35531<br>12 Diabetes, Gestational/ 12317<br>13 Hypertension, Pregnancy-Induced/ 3775<br>14 exp Urinary Incontinence/ 34163<br>15 Pelvic Organ Prolapse/ 4269<br>16 (perinatal or postpartum or postnatal).mp. [mp=title, abstract, original title, name of substance word, subject heading word, floating sub-heading word, keyword heading word, organism supplementary concept word, protocol supplementary concept word, rare disease supplementary concept word, unique identifier, synonyms] 247345<br>17 7 or 8 or 9 or 10 or 11 or 12 or 13 or 14 or 15 or 16 312631<br>18 "Attitude of Health Personnel"/ or Attitude/ 176055<br>19 Perception/ 38727<br>20 (attitude* or belief* or opinions* or understanding* or viewpoint* or perspective* or perception* or view* or experience*).mp. [mp=title, abstract, original title, name of substance word, subject heading word, floating sub-heading word, keyword heading word, organism supplementary concept word, protocol supplementary concept word, rare disease supplementary concept word, unique identifier, synonyms] 3341799<br>21 18 or 19 or 20 3341799<br>22 6 and 17 and 21 649<br>Limited to 1990-2021 = 607
**PsychInfo**	APA PsycInfo <1967 to September Week 3 2021><br><br>1 General Practitioners/ 6045<br>2 Primary Health Care/ 19408<br>3 Physicians, Primary Care/ 0<br>4 Physicians, Family/ 0<br>5 (general practitioner* or GP*).mp. [mp=title, abstract, heading word, table of contents, key concepts, original title, tests & measures, mesh] 27828<br>6 1 or 2 or 3 or 4 or 5 44630<br>7 Perinatal Care/ 0<br>8 Postpartum Period/ 0

Supplementary File 1: Search Terms

	9 Postnatal Care/ 0<br>10 Maternal Health/ 0<br>11 exp Puerperal Disorders/ 0<br>12 Diabetes, Gestational/ 0<br>13 Hypertension, Pregnancy-Induced/ 0<br>14 exp Urinary Incontinence/ 2004<br>15 Pelvic Organ Prolapse/ 0<br>16 (perinatal or postpartum or postnatal).mp. [mp=title, abstract, heading word, table of contents, key concepts, original title, tests & measures, mesh] 42320<br>17 7 or 8 or 9 or 10 or 11 or 12 or 13 or 14 or 15 or 16 44299<br>18 "Attitude of Health Personnel"/ or Attitude/ 0<br>19 Perception/ 19909<br>20 (attitude* or belief* or opinions* or understanding* or viewpoint* or perspective* or perception* or view* or experience*).mp. [mp=title, abstract, heading word, table of contents, key concepts, original title, tests & measures, mesh] 1955569<br>21 18 or 19 or 20 1955569<br>22 6 and 17 and 21140<br>23 from 22 keep 1-3,5-137 136Limited to 1990 – 2021 = 136 (LIMITED TO 1990-2021)
**EMBASE**	Embase <1974 to 2021 September 27><br>1 General Practitioners/ 100397<br>2 Primary Health Care/ 71097<br>3 Physicians, Primary Care/ 106008<br>4 Physicians, Family/ 100399<br>5 (general practitioner* or GP*).mp. [mp=title, abstract, heading word, drug trade name, original title, device manufacturer, drug manufacturer, device trade name, keyword heading word, floating subheading word, candidate term word] 381855<br>6 1 or 2 or 3 or 4 or 5 444488<br>7 Perinatal Care/ 14860<br>8 Postpartum Period/ 35277<br>9 Postnatal Care/ 7813<br>10 Maternal Health/ 15058<br>11 exp Puerperal Disorders/ 45592<br>12 Diabetes, Gestational/ 19790<br>13 Hypertension, Pregnancy-Induced/ 11617<br>14 exp Urinary Incontinence/ 79211<br>15 Pelvic Organ Prolapse/ 15877<br>16 (perinatal or postpartum or postnatal).mp. [mp=title, abstract, heading word, drug trade name, original title, device manufacturer, drug manufacturer, device trade name, keyword heading word, floating subheading word, candidate term word] 336868<br>17 7 or 8 or 9 or 10 or 11 or 12 or 13 or 14 or 15 or 16 483411<br>18 "Attitude of Health Personnel"/ or Attitude/ 138747<br>19 Perception/ 135053<br>20 (attitude* or belief* or opinions* or understanding* or viewpoint* or perspective* or perception* or view* or experience*).mp. [mp=title, abstract, heading word, drug trade name, original title, device manufacturer, drug

Supplementary File 1: Search Terms

	manufacturer, device trade name, keyword heading word, floating subheading word, candidate term word] 4291704<br>21 18 or 19 or 20 4291704<br>22 6 and 17 and 21 1265<br>Limited to 1990-2021 = 1228
**Web of Science**	Timespan: 1990-01-01 to 2021-09-28 (Index Date)<br><br>'General Practitioner*' OR 'Primary Health Care' OR 'Family Physician*' OR 'GP*' and 'perinatal care' OR 'postpartum period' OR postnatal care' OR 'maternal health' OR 'puerperal disorders' OR 'diabetes, gestational' OR 'pelvic organ prolapse' OR 'perinatal' or 'postpartum' OR 'postnatal' and 'perception*' OR 'attitude*' OR 'belief*' OR 'opinion*' OR 'understanding' OR viewpoint*' OR 'perspective*' OR 'perception*' OR 'view*' OR 'experience*'<br>=223
**PubMed**	("general practitioners"[MeSH Terms] OR "primary health care"[MeSH Terms] OR "physicians, primary care"[MeSH Terms] OR "physicians, family"[MeSH Terms]OR 'general practitioner' OR 'GP') AND ("perinatal care"[MeSH Terms] OR "postpartum period"[MeSH Terms] OR "postnatal care"[MeSH Terms] OR "maternal health"[MeSH Terms] OR "puerperal disorders"[MeSH Terms] OR "diabetes, gestational"[MeSH Terms] OR "hypertension, pregnancy induced"[MeSH Terms] OR "urinary incontinence"[MeSH Terms] OR "pelvic organ prolapse"[MeSH Terms] OR 'perinatal' OR 'postpartum' OR 'postnatal') AND ("attitude"[MeSH Terms] OR "attitude of health personnel"[MeSH Terms] OR "perception"[MeSH Terms] OR attitude OR belief OR opinion OR understanding OR viewpoint OR perspective OR perception OR view OR experience)<br>= 1743<br><br>Limited to 1990 – 2021 = 1662
**CINAHL**	((MH "Physicians, Family") OR "general practitioner" OR "GP") AND ((MH "Postpartum Care (Saba CCC)") OR (MH "Postnatal Care") OR (MH "Postnatal Period") OR (MH "Depression, Postpartum") OR (MH "Postpartum (Omaha)") OR (MH "Puerperium") OR "("perinatal care" OR "postpartum period" OR "postnatal care" OR "maternal health" OR "puerperal disorders" OR "diabetes, gestational" OR "hypertension, pregnancy induced" OR "urinary incontinence" OR "pelvic organ prolapse" OR 'perinatal' OR 'postpartum' OR 'postnatal'" OR (MH "Perinatal Care (Saba CCC)") OR (MH "Urinary Incontinence Care (Saba CCC)") OR (MH "Perinatal Care") OR (MH "Diabetes Mellitus, Gestational") OR (MH "Pelvic Organ Prolapse") OR (MH "Pregnancy-Induced Hypertension")) AND ("'attitude*' 'belief*' 'opinion*' 'understanding*' 'viewpoint*' 'perspective*' 'perception*' 'view*' 'experience*'" OR (MH "Attitude of Health Personnel") OR (MH "Physician Attitudes") OR (MH "Attitude to Mental Illness") OR (MH "Attitude to Breast Feeding") OR (MH "Caregiver Attitudes") OR (MH "Attitude to Medical Treatment") OR (MH "Job Experience") OR (MH "Perception") OR (MH "Attitude"))
