# Supplementary material for: Qualitative systematic review of general practitioners’ (GPs’) views and experiences of providing postnatal care
Source: BMJ Open. 2023 Apr 11;13(4):e070005. doi: 10.1136/bmjopen-2022-070005 (PMC10106050; doi:10.1136/bmjopen-2022-070005)
Supplement: Supplementary data [file bmjopen-2022-070005supp002.pdf]

Summary of study characteristics

Title	Author	Year	Setting	Recruitment	Number of GPs	Additional participants	Focus of study	Data collection from GPs	Analysis methodology	Comments
**Postnatal care following hypertensive disorders of pregnancy: a qualitative study of views and experiences of primary and secondary clinicians**	Bick et al	2019	UK	GPs invited from practices in 3 London maternity unit areas	5	Obstetricians, physician, health visitors, midwives (n=15)	Views of clinicians caring for postnatal women who had HDP	Semi-structured interviews	Thematic	
**The views of mothers and GPs about postpartum care in Australian general practice**	Brodribb et al	2013	Australia	GPs in locations serving hospitals taking part in a postnatal care study	6	Mothers (n=88)	Views about postpartum care in general practice	Interviews	Thematic	Reports on its pilot, and larger study together. Only one theme reported in full, authors contacted but no response
**GPs' and health visitors' views on the diagnosis and management of postnatal depression**	Chew-Graham et al	2008	UK	Invitation via practices who were participating in an associated RCT. Purposive by GP demographics.	19	Health visitors (n=14)	Views on role in detection and management of postnatal depression	In-depth interviews	Thematic	
**Disclosure of symptoms of postnatal depression, the perspectives of health professionals and women: a qualitative study**	Chew-Graham et al	Reports on same study as above								
**Investigating the current knowledge and needs concerning follow-up for long-term cardiovascular risks in Dutch women with a preeclampsia history: a qualitative study**	Dijkhuis et al	2020	Netherlands	Convenience, GP offices in region of hospital study base	4	Women, obstetricians, vascular medicine specialists, cardiologists (n=25)	Opinions and wishes about follow-up for women with a history of pre-ecplampsia	Semi-structured interviews	Thematic	
**Falling through the net - Black and minority ethnic women and perinatal mental healthcare: health professionals' views**	Edge	2010	UK	GPs from areas with high Black Caribbean population	5	Midwives, hospital doctors, health visitors (n=37)	Investigating views about perinatal mental healthcare for BAME women	In-depth interviews	Framework	
**Health Care Providers' Perceptions of Responsibilities and Resources to Reduce Type 2 Diabetes Risk After Gestational Diabetes Mellitus**	Hewage et al	2018	Singapore	Purposive, snowball sampling	5	OB/GYN, endocrinologists, dietitians, nurses (n=27)	Healthcare providers' perceptions of care responsibilities and resources related to reducing T2DM among women with previous GDM	In-depth interviews	Framework	
**GPs' decision-making when prescribing medicines for**	Jayawickrama et al	2010	Australia	Anonymous postal questionnaire	253	-	Factors influencing GPs' decision-making when	Survey	Thematic Networks	

**breastfeeding women: Content analysis of a survey**							prescribing for breastfeeding women			
**Falling through the gaps: perinatal mental health and general practice**	Khan	2018	UK	Convenience (large scale dissemination through virtual portals)	43	Women (n=1551)	Experiences of interactions between women with common perinatal mental health problems and GPs	Survey and semi-structured interviews	Interpretative phenomenolo gical approaches	
**Postnatal gestational diabetes mellitus follow-up: Perspectives of Australian hospital clinicians and general practitioners**	Kilgour et al	2018	Australia	GPs who worked in collaboration with a large city hospital (where the hospital clinicians were recruited from)	16	Hospital clinicians (n=13)	Communication processes between GPs and hospital clinicians providing postnatal GDM care		Computer automated Leximancer analysis	
**Experiences of women, hospital clinicians and general practitioners with gestational diabetes mellitus follow-up: A mixed methods approach**	Kilgour et al	Reports on same study as above, but with 2 further (quant) stages. Some additional GP qual data included in this publication to be included.								
**Views of general practitioners on providing contraceptive advice and long-acting eversible contraception at the 6-week postnatal visit: a qualitative study**	Lunniss et al	2015	UK	Purposive (practice demographics), , convenience (education event), snowball	13	-	Views on contraception provision including LARCs at 6 week check	Semi-structured interviews	Thematic	
**An exploratory qualitative study exploring GPs' and psychiatrists' perceptions of post-traumatic stress disorder in postnatal women using a fictional case vignette**	Mortimer et al	2021	UK	Email invitation / poster adverts (not explicit whether purposive)	6	Psychiatrists (n=7)	GP and psychiatrist perceptions of PTSD in postnatal women	Semi-structured interviews with a case vignette	Framework	
**Irish general practitioners' view of perinatal mental health in general practice: a qualitative study**	Noonan et al	2018	Ireland	Purposive, GPs connected with GP tutors from	10	-	Caring for women with perinatal mental illness	Semi-structured interviews	Thematic	
**Improving follow-up care for women with a history of gestational diabetes: perspectives of GPs and patients**	Pennington et al	2017	Australia	Purposive (GP and practice demographics)	18	Women (n=16)	Perspectives of factors influencing engagement with diabetes prevention after a pregnancy with GDM	Semi-structured interviews	Thematic content	
**Experiences of primary care physicians managing postpartum care: a qualitative research study**	Poon et al	2021	Singapore	Purposive (demographics), networks of the researchers	29	-	Managing postpartum women	Focus Groups In depth interviews	?thematic, unclear	
**Healthcare professionals' perspectives on identifying and managing perinatal anxiety: a qualitative study**	Silverwood et al	2019	UK	GPs via WM-CRN and personal contacts	10	Midwives, HVs, (n=12)	Identification and management of perinatal anxiety	Semi-structured interviews	Constant comparison	

**Towards an understanding of GPs' viewpoint on diagnosing postnatal depression in general practice: a small-scale realist evaluation**	Sriranjan et al	2020	UK	Convenience sample, single GP practice	7	-		Semi-structured interviews with case vignettes	Framework analysis	
**Behaviour change opportunities at mother and baby checks in primary care: a qualitative investigation of the experiences of GPs**	Talbot et al	2018	UK	Snowball sampling GP practices	18	-	Health behaviour change opportunities at the postnatal check	Semi-structured interviews - phone	Thematic analysis	
**Healthcare Professionals' Perspectives on the Cross-Sectoral Treatment Pathway for Women with Gestational Diabetes during and after Pregnancy – A Qualitative Study**	Timm et al	2021	Denmark	Purposive to cover antenatal and postnatal period, by email / phone (selection not clearly stated)	2	Midwives, obstetricians, nurse, health visitors (n=15)	Management of GDM in and after pregnancy	Semi-structured interviews	Systematic Text Condensation	
