# Supplementary material for: Qualitative systematic review of general practitioners’ (GPs’) views and experiences of providing postnatal care
Source: BMJ Open. 2023 Apr 11;13(4):e070005. doi: 10.1136/bmjopen-2022-070005 (PMC10106050; doi:10.1136/bmjopen-2022-070005)
Supplement: Supplementary data [file bmjopen-2022-070005supp003.pdf]

Supplementary File 3: CASP Assessment Table

	Bick et al (2019)	*Brodribb et al (2013)*	*Chew-Graham et al (2008)*	*Dijkhuis et al (2020)*	*Edge et al (2010)*	*Hewage et al (2018)*	*Jayawickrama et al (2010)*	*Kham (2015)*	*Kilgour et al (2018*	*Lunniss et al (2015)*	*Mortimer et al (2021)*	*Noonan et al (2018)*	*Pennington et al (2017)*	*Poon et al (2021)*	*Silverwood et al (2019)*	*Sriranjan et al (2020)*	*Talbot et al (2018)*	*Timm et al (2021)*
**Was there a clear statement of the aims of the research?**	Y	Y	Y	Y	Y	Y	Y	N	Y	Y	Y	Y	Y	Y	Y	Y	Y	Y
**Is a qualitative methodology appropriate?**	Y	Y	Y	Y	Y	Y	Y	Y	Y	Y	Y	Y	Y	Y	Y	Y	Y	Y
**Was the research design appropriate to address the aims of the research?**	Y	Y	Y	Y	Y	Y	Y	Unclear	Y	Y	Y	Y	Y	Y	Y	Y	Y	Y
**Was the recruitment strategy appropriate to the aims of the research?**	Y	Y	Y	Y	Y	Y	Y	N	Y	Y	Y	Y	Y	Y	Y	Y	Y	Y
**Was the data collected in a way that addressed the research issue?**	Y	Y	Y	Y	Y	Y	N	Y	Y	Y	Y	Y	Y	Y	Y	Y	Y	Y
**Has the relationship between researcher and participants been adequately considered?**	Unclear	N	N	Y	N	N	N	N	Y	N	Y	Y	N	Y	N	Y	N	N
**Have ethical issues been taken into consideration?**	Y	Y	Y	Y	Y	Y	Y	Unclear	Y	Y	Y	Y	Y	Y	Y	Y	Y	Y
**Was the data analysis sufficiently rigorous?**	Y	Y	Y	Y	Y	Y	Y	N	Y	N	Y	Y	Y	Y	Y	Y	Y	Y
**Is there a clear statement of findings?**	Y	Y	Y	Y	Y	Y	Y	Y	Y	Y	Y	Y	Y	Y	Y	Y	Y	Y
**How valuable is the research; will the results help locally?**	Y	Y	Y	Y	Y	N	Y	Y	Y	Y	Y	Y	Y	Y	Y	Y	Y	Y
