# Supplementary material for: Qualitative systematic review of general practitioners’ (GPs’) views and experiences of providing postnatal care
Source: BMJ Open. 2023 Apr 11;13(4):e070005. doi: 10.1136/bmjopen-2022-070005 (PMC10106050; doi:10.1136/bmjopen-2022-070005)
Supplement: Supplementary data [file bmjopen-2022-070005supp004.pdf]

Supplementary File 4: Descriptive Themes

Descriptive Theme	Description / definition
Content and timing of postnatal checks	Any reference to content or timing of postnatal reviews, including follow-up or monitoring of pregnancy-specific pathology
Agenda setting - woman or GP	GPs' beliefs about what women want to address, or references to GPs deciding what they will cover
Baby as distraction	Baby issues taking priority, focus being baby-driven postnatally
Clinical decision making	Beliefs about diagnoses and clinical management, descriptions about how pathology is managed
Communication between other services and primary care	Communication from hospital to primary care, or other services and primary care, including notification of which patients need specific follow-up etc
Continuity of care	Specific mentions of continuity of care – either services separate from primary care (e.g. views on midwifery continuity of care) or GP views on GP continuity of care
Doctor-related factors and personal experience	Characteristics / descriptive statements about doctors that influence (positive or negative) care including personal experience
Guidelines, screening tools and prompts	Including use of guidelines, prompts or screening tools and references to their use
Knowledge	What GPs say they know, or do not know
Organisation of primary care and health visiting	GP services, including access e.g. booking appointments, arranging follow-up and descriptions about how HV is organised, including how it has changed
Referral to other services	Including accessing patient-specific advice
Role of the GP and MDT members	Descriptions of the nature of the role of the GP and other healthcare professionals e.g. HV / pharmacists / midwives
Time-related factors	Including duration of the appointment, issues that impact time pressures
Training and education	References to training received, or training wanted
Woman-related factors	GPs views on factors about women that influences care, including involvement (or not) of their partners
