# Supplementary material for: Qualitative systematic review of general practitioners’ (GPs’) views and experiences of providing postnatal care
Source: BMJ Open. 2023 Apr 11;13(4):e070005. doi: 10.1136/bmjopen-2022-070005 (PMC10106050; doi:10.1136/bmjopen-2022-070005)
Supplement: Supplementary data [file bmjopen-2022-070005supp005.pdf]

Mapping descriptive themes to the COM-B domains

COM-B domain	Descriptive Themes	Sources
**Psychological Capability**	Knowledge	(31, 35, 40, 41, 44, 47, 49, 50)
**Physical Capability**	None	
**Physical Opportunity**	Time-related factors<br>Screening tools or prompts<br>Organisation of primary care and health visiting<br>Referral to other services<br>Continuity of care<br>Communication between services<br>Training and education<br>Clinical guidelines, screening tools or prompts	(31-50)
**Social Opportunity**	Woman-related factors<br>Baby as distraction<br>Antenatal and intrapartum experiences<br>Agenda setting<br>GP roles<br>MDT roles	(31-42, 44-46, 48-50)
**Reflective and Automatic Motivation**	Doctor-related factors and personal experience<br>Clinical decision making	(32-34, 37, 38, 39, 43-49)
