# Supplementary material for: Qualitative systematic review of general practitioners’ (GPs’) views and experiences of providing postnatal care
Source: BMJ Open. 2023 Apr 11;13(4):e070005. doi: 10.1136/bmjopen-2022-070005 (PMC10106050; doi:10.1136/bmjopen-2022-070005)
Supplement: Author's manuscript [file bmjopen-2022-070005.draft_revisions.pdf]

BMJ Open

BMJ Open is committed to open peer review. As part of this commitment we make the peer review history of every article we publish publicly available.

When an article is published we post the peer reviewers' comments and the authors' responses online. We also post the versions of the paper that were used during peer review. These are the versions that the peer review comments apply to.

The versions of the paper that follow are the versions that were submitted during the peer review process. They are not the versions of record or the final published versions. They should not be cited or distributed as the published version of this manuscript.

BMJ Open is an open access journal and the full, final, typeset and author-corrected version of record of the manuscript is available on our site with no access controls, subscription charges or pay-per-view fees (http://bmjopen.bmj.com).

If you have any questions on BMJ Open's open peer review process please email info.bmjopen@bmj.com

BMJ Open

A qualitative systematic review of general practitioners' (GPs') views and experiences of providing postnatal care.

Journal:	*BMJ Open*
Manuscript ID	bmjopen-2022-070005
Article Type:	Original research
Date Submitted by the Author:	11-Nov-2022
Complete List of Authors:	Macdonald, Clare; University of Birmingham, Institute of Applied Health Research<br>MacGregor, Becky; University of Warwick, Unit of Academic Primary Care<br>Hillman, Sarah ; University of Warwick, Unit of Academic Primary Care<br>MacArthur, Christine; University of Birmingham, Institute of Applied Health Research<br>Bick, Debra; University of Warwick<br>Taylor, Beck; University of Birmingham, Institute of Applied Health Research
Keywords:	REPRODUCTIVE MEDICINE, QUALITATIVE RESEARCH, PRIMARY CARE, Maternal medicine < OBSTETRICS, Reproductive medicine < GYNAECOLOGY

SCHOLARONE™
Manuscripts

I, the Submitting Author has the right to grant and does grant on behalf of all authors of the Work (as defined in the below author licence), an exclusive licence and/or a non-exclusive licence for contributions from authors who are: i) UK Crown employees; ii) where BMJ has agreed a CC-BY licence shall apply, and/or iii) in accordance with the terms applicable for US Federal Government officers or employees acting as part of their official duties; on a worldwide, perpetual, irrevocable, royalty-free basis to BMJ Publishing Group Ltd ("BMJ") its licensees and where the relevant Journal is co-owned by BMJ to the co-owners of the Journal, to publish the Work in this journal and any other BMJ products and to exploit all rights, as set out in our licence.

The Submitting Author accepts and understands that any supply made under these terms is made by BMJ to the Submitting Author unless you are acting as an employee on behalf of your employer or a postgraduate student of an affiliated institution which is paying any applicable article publishing charge ("APC") for Open Access articles. Where the Submitting Author wishes to make the Work available on an Open Access basis (and intends to pay the relevant APC), the terms of reuse of such Open Access shall be governed by a Creative Commons licence – details of these licences and which Creative Commons licence will apply to this Work are set out in our licence referred to above.

Other than as permitted in any relevant BMJ Author's Self Archiving Policies, I confirm this Work has not been accepted for publication elsewhere, is not being considered for publication elsewhere and does not duplicate material already published. I confirm all authors consent to publication of this Work and authorise the granting of this licence.

For peer review only

A qualitative systematic review of general practitioners' (GPs') views and experiences of providing postnatal care.

Authors

1. Clare Macdonald. Institute of Applied Health Research, University of Birmingham, Birmingham, B15 2TT. UK. CCM011@student.bham.ac.uk (corresponding author)
2. Becky MacGregor. Unit of Academic Primary Care, University of Warwick, Coventry, CV4 7AL. UK.
3. Sarah Hillman. Unit of Academic Primary Care, University of Warwick, Coventry, CV4 7AL. UK.
4. Christine MacArthur. Institute of Applied Health Research, University of Birmingham, Birmingham, UK. B15 2TT.
5. Debra Bick. University of Warwick, Coventry, West Midlands, CV4 7AL. UK.
6. Beck Taylor. Institute of Applied Health Research, University of Birmingham, Birmingham, UK. B15 2TT.

Word count (excluding title page, abstract, references, figures and tables): 4889

Abbreviations: CM - Clare Macdonald, BM - Becky MacGregor, SH - Sarah Hillman, BT - Beck Taylor

Author contributions: All authors contributed to the design, study selection, analysis and writing and approved the final manuscript.

PROSPERO registration: CRD42021268982

Funding statement: Clare Macdonald is funded by the National Institute for Health Research (NIHR) West Midlands Applied Research Collaboration. The views expressed are those of the author and not necessarily those of the NIHR or the UK Department of Health and Social Care.

Where we use the terms 'woman' or 'women' in the context of their postnatal care, this is inclusive of all people who have given birth even if they do not identify as women.

For peer review only - http://bmjopen.bmj.com/site/about/guidelines.xhtml

ABSTRACT

Objectives

Develop an understanding of the views and experiences of GPs about their role in postnatal care, including barriers and facilitators to good care, and timing and content of planned postnatal checks.

Design

Qualitative systematic review.

Data sources

Electronic database searches of MEDLINE, EMBASE, CINAHL, PubMed, Web of Science, PsychINFO. Grey literature and guideline references from National Institute of Health and Care Excellence, World Health Organization, International Federation of Gynecology and Obstetrics, Royal College of General Practitioners, Royal College of Obstetrics and Gynaecology.

Inclusion criteria

Papers reporting qualitative data on views and experiences of GPs about postnatal care, including discrete clinical conditions in the postnatal period. Papers were screened independently by two reviewers and disputes resolved by a third reviewer.

Quality appraisal

The CASP (Critical Appraisal Skills Programme) checklist was used to appraise studies.

Data extraction and synthesis

Thematic synthesis involving line-by-line coding, generation of descriptive then analytical themes was conducted by the review team. The COM-B model was used to develop analytical themes.

Results

20 reports from 18 studies met inclusion criteria. Studies were published from 2008-2021, reporting on 469 GPs. 13 were from UK or Australia. Some also reported views of non-GP participants. The clinical focus of studies varied, for example: perinatal mental health, postnatal contraception. Five themes were generated, four mapped to COM-B: psychological capability, physical opportunity, social opportunity and motivation. One theme was separate from the COM-B model: content and timing of postnatal checks. Strong influences were in physical and social opportunity, with time and organisation of services being heavily represented. These factors sometimes influenced findings in the motivation theme.

Conclusions

GPs perceived their role in postnatal care as a positive opportunity for relationship building and health promotion. Addressing organisational barriers could impact positively on GPs' motivation to provide the best care.

For peer review only - http://bmjopen.bmj.com/site/about/guidelines.xhtml

For peer review only

STRENGTHS AND LIMITATIONS

- This review synthesised the views and experiences of GPs about postnatal care, including its entire remit
- The search strategy was comprehensive, including grey literature and citation searches
- Only reports in English were included so some potentially relevant studies could have been overlooked
- Some included studies reported on non-GP participants in addition to GPs and it was not always possible to be certain which data was attributable to GPs
- Heterogeneity of study focus was high, and the analysis therefore synthesised the views of GPs across multiple aspects of postnatal care (for example, postnatal contraception and gestational diabetes) without certainty that these are applicable to every aspect

For peer review only

BACKGROUND

UK primary health care is generally organised and provided by practices who operate as independent contractors to the National Health Service (NHS), delivering 'General Medical Services'(1) to their registered patients. Since 2020, as part of this contract, providers in England are required to offer all women a postnatal check appointment 6-8 weeks after birth.(2) Community postnatal care, at home following birth, is multidisciplinary; as a minimum, midwives, health visitors and general practitioners (GPs) are typically involved. GPs see women for unplanned care in the postnatal period and for the routine 6–8-week check. Historically, in the UK, postnatal care provided after discharge from hospital has been an area of low priority in maternity services. Successive reports have found that women's experiences are often poor,(3,4) measurable outcomes such as breastfeeding rates are low(5) and even national guidance contextualises recommendations by describing postnatal care as a 'Cinderella subject'.(6) This is in spite of the postnatal period being a high risk time for maternal mortality and the unplanned maternal readmission rate being around 3.3%.(7)

A multidisciplinary, community-based postnatal care model is not unique to the UK. A systematic review of guidelines of community postnatal care found comparable recommendations from Australia and USA.(8) More recently, Canadian public health guidance included similar principles and themes and, similar to UK, stated that multiple healthcare professionals will be involved with postpartum care.(9) In its position statement from 2021, the Royal Australian College of General Practitioners stated that there were gaps in evidence for the role of GPs in maternity care with more research needed.(10)

UK GPs have become less involved in routine maternity care over time, markedly declining since the 1990s when intrapartum involvement dramatically reduced.(11) GPs are now often absent from antenatal care with women self-referring to NHS midwifery services, and midwives providing antenatal care even for women who subsequently require an obstetrician.(12) GPs have generally continued seeing women routinely around 6-8 weeks postnatally even when from 2004-2020 they had no contractual obligation to do so.(13) The quality was, however, variable; a 2017 report found women often felt rushed and 42% of those with emotional or mental health problems did not have this identified by their GP or other health professional.(14) This report called for designated funding for the GP postnatal check and the Royal College of General Practitioners (RCGP) echoed this.(15) In 2020, the review of the NHS England GP contract included specific funding for the GP 6-8 week.(2) Soon after this, the National Institute for Health and Care Excellence (NICE) published its updated Postnatal Care Guideline(6) specifying that the check should be done by a GP.

For peer review only - http://bmjopen.bmj.com/site/about/guidelines.xhtml

The volume and variety of topics that should be covered in the maternal postnatal check, according to NICE is vast, even with no patient-related complexity. Factors such as mode of birth, intrapartum complications and co-morbidities can affect the content of the consultation. Crucial to informing GPs' knowledge and understanding of the complexity of women's postnatal care requirements is information about their antenatal and intrapartum health and care provided by the hospital. NICE recommends that communication between secondary (i.e. hospital) and primary care is prompt and effective, including a plan for ongoing care and conditions that require long-term management. Current arrangements for the maternal postnatal check do not distinguish formally between women who require more complex, or additional follow-up due to health and/or potential social care complications from those who do not.

To the authors' knowledge, no previous review has evaluated the views and experiences of GPs on postnatal care, incorporating the various clinical topics that form the 6-8 week maternal check as defined by NICE. There are examples of qualitative systematic reviews on discrete postnatal care topics, for example gestational diabetes(16) and promotion of a healthy lifestyle in postpartum women,(17) but these do not provide an in-depth qualitative synthesis of the views and experiences of GPs encompassing the whole remit of postnatal care. The Cochrane Collaboration have planned a qualitative systematic review of factors influencing the provision of postnatal care by health workers,(18) but this will also include secondary and tertiary care settings with no specific focus on GPs' perspectives. Women's experiences are better documented than GPs' experiences and suggest inconsistencies in provision of care.(19) Given the broad scope and variability in quality of the GP 6-8 week maternal postnatal check, in order to understand the dynamic of these consultations and potential practitioner-based facilitators or barriers, it is necessary to understand the contextual and behavioural basis that potentially impacts the provision of postnatal care by GPs.

AIMS

This qualitative systematic review aimed to answer four research questions:

1. What do GPs say is, or should be, the content of the routine 6-8 week check (including non-clinical topics such as social complexity)
2. At what point in time do GPs say that they routinely review women after birth, and what timing do they think is optimal?
3. What do GPs see as the facilitators and barriers to providing high quality postnatal care in primary care settings?
4. What do GPs perceive to be their role in postnatal care, and what role do they say other members of the primary care team have, or should have?

For peer review only - http://bmjopen.bmj.com/site/about/guidelines.xhtml

METHODS

The review protocol was registered on the international prospective register of systematic reviews (PROSPERO).

Inclusion and exclusion criteria

The PerSPecTIF framework(20) was used to develop and define the detail of the research questions, and therefore eligibility criteria for inclusion of studies (Table 1). Given variability in provision of postnatal care in primary care, particularly the postnatal check, this framework was chosen with the aim of achieving a better understanding of context and acceptability from the perspectives of individuals about care provision. There was no intervention evaluated, or comparison made.

For peer review only

For peer review only - http://bmjopen.bmj.com/site/about/guidelines.xhtml

Table 1: PerSPecTIF Table of inclusion and exclusion criteria

PerSPecTIF	Inclusion	Comments
**Per**spective	General practitioners<br>General practitioner specialty trainees (GPSTs) (sometimes termed registrars)<br>Primary care physicians	Exclude hospital physicians / obstetricians / paediatricians<br>Exclude other healthcare workers e.g. midwives, health visitors, pharmacists
**S**etting	Primary care	Exclude secondary care settings
**P**henomenon of interest	Postnatal care of women and routine postnatal checks for women	Including the following defined clinical aspects of postnatal care<br>- Postnatal contraception<br>- Postnatal mental illness<br>- Gestational diabetes<br>- Hypertensive disorders of pregnancy<br>- Urinary incontinence<br>- Pelvic organ prolapse<br>Including, but not limited to other relevant topics such as postnatal infections (mastitis, perineal wound infection, caesarean section wound infection), breastfeeding related problems.<br>Including health promotion, social issues or concerns, social complexity, social prescribing
**E**nvironment	Community settings providing planned (routine review or follow-up) and unplanned postnatal care from any GPs	
**(C)**omparison	No comparison	
**T**iming	The postnatal period, encompassing the first 2 years after giving birth (because perinatal mental health services accept referrals up until 2 years after birth)	
**F**indings	In relation to the views and experiences of GPs, with relevance to GPs, policy makers, medical educators	

For peer review only - http://bmjopen.bmj.com/site/about/guidelines.xhtml

Information Sources

Database searches of MEDLINE, EMBASE, CINAHL, PubMed, Web of Science and PsychINFO were carried out in September 2021. Reference lists of included studies were reviewed for other potential studies meeting inclusion criteria, as were references from relevant grey literature including guidance from NICE, World Health Organization (WHO), International Federation of Gynecology and Obstetrics (FIGO), RCGP and Royal College of Obstetrics and Gynaecology (RCOG). Citation searching of included publications was completed. Grey literature and citation searches were conducted in March 2022.

Search Strategy

Searches were limited to publications where full text was available in English. Studies from before 1990 were excluded since UK GP involvement in maternity services became more confined to postnatal care from that time. Studies with populations from low and middle income countries(21) were excluded to maintain relevance to UK setting. The search strategy was piloted to ensure it captured key studies known to reviewers. (see supplementary file : Supp1)

Screening and data extraction

After removal of duplicates, titles and abstracts of all records were independently screened by two reviewers (CM and SH or BM). A third reviewer (BT) screened records where there was disagreement. A standardised data extraction proforma was developed to extract consistent, relevant information from studies including year of publication, setting, and analysis methodology. Data were extracted in line with the approach described by Thomas and Harden(22) whereby anything reported as 'findings', 'results' or equivalent were included in the analysis. Data from non-GP participants were not extracted. Data included themes, descriptions of themes and participant quotes. Full texts, including abstracts were checked for anything that could be considered a study finding. CM and SH or BM independently completed data extraction for a third of the included studies and there was a high degree of consistency, CM then completed data extraction for the remaining studies.

Data Synthesis

The thematic synthesis approach outlined by Thomas and Harden(22) was used as the basis for data synthesis. After multiple readings to gain familiarity, CM imported extracted data into NVivo12 software. Line by line coding was undertaken by CM, with BT independently coding a third of data and consistency of approach confirmed. CM and BT, in discussion with other review team members developed descriptive codes which was inductive, but with reference to the research questions. The data synthesis process was iterative and collaborative, taking place over several weeks with continual reference to the original studies and input from the review team to ensure that interpretation reflected the original studies. The review team remained open to the possibility of building the

For peer review only - http://bmjopen.bmj.com/site/about/guidelines.xhtml

analytical themes around pre-existing frameworks or models, depending on the nature of the data and the descriptive themes identified.

RESULTS

Included studies

2693 unique references were identified in database searches and following screening, 23 full-text reports were assessed for eligibility by CM. The review team agreed the final set of included studies. 17 reports describing 15 studies met inclusion criteria from this process. One excluded report was an integrative review on physician perspectives of their role in perinatal mental health.(23) Studies included in this report were screened and two of these met eligibility criteria. Reasons for exclusion from the database searches were incorrect phenomenon of interest(24, 25) or incorrect perspective.(26-30) Three studies from references or citations of eligible reports met the inclusion criteria and were also included. In total, 20 reports were eligible, reporting on 18 studies (Figure 1).(31-50)

Studies were published between 2008 and 2021 representing views and experiences of 469 GPs. Nine were UK studies, four Australian, and the remainder from other countries, all with some degree of similarity to the UK postnatal care model. Twelve studies included other clinicians and GPs (e.g. health visitors, obstetricians), six included GPs only. As anticipated, studies focused on a range of postnatal care topics, or postnatal care overall. Most frequent were perinatal mental health (n=7) and follow-up of gestational diabetes (n=4). The remainder investigated postnatal care generally, follow-up of hypertension or pre-eclampsia, behaviour change opportunities, contraception and prescribing for breastfeeding women. Two studies reported qualitative analysis of survey results, others used various forms of interview for data collection; the inclusion of qualitative analysis of survey data explains the large number of participants overall. (see supplementary file: Supp2)

Quality appraisal

Quality appraisal was undertaken using the Critical Skills Appraisal Programme (CASP) Qualitative Checklist.(51) Studies were not excluded on methodological merit, but CASP was used to formally understand their integrity and reliability which aided interpretation of findings during the synthesis. CM completed the CASP assessment, with oversight from the review team. Most studies fulfilled all or nearly all the checklist criteria, the area with least compliance being consideration of the relationship between researcher and participants which was only fulfilled in five studies. (see supplementary file: Supp3)

Themes

For peer review only - http://bmjopen.bmj.com/site/about/guidelines.xhtml

16 descriptive themes were identified (see supplementary file: Supp4). The final stage of thematic synthesis involved identifying analytical themes; moving beyond a descriptive summary of aggregated findings. One descriptive theme related specifically to two of the research questions – content and timing of the postnatal check. This did not generate new, analytical findings and is summarised descriptively. The remainder of the descriptive themes related to influences, barriers and facilitators to GPs in providing postnatal care. When reviewing and discussing these descriptive themes, to move to analytical themes, the research team reflected that these factors could in fact be mapped to the COM-B (Capability, Opportunity, Motivation – Behaviour) model; (52) (Figure 2) this became the basis upon which the final analytical stage was completed. The descriptive themes were reviewed with reference to COM-B and mapped to relevant aspect of the model. The final themes were therefore: content and timing of postnatal checks (not mapped to COM-B), psychological capability, physical opportunity, social opportunity and motivation (no descriptive themes were mapped to the COM-B domain of 'physical capability'). (Figure 3). (see supplementary file: Supp5)

Content and timing of postnatal checks

Most studies mentioned the content of a routine postnatal check, or the typical remit of GPs' interactions with women.(31, 32, 35-37, 39-42, 44-46, 48-50) A broad range of specific clinical topics were cited as falling under GP responsibility, including asking about complications, such as hypertension or diabetes, more universal issues such as mental health, bleeding, pain, wound healing, fever, breastfeeding, sleep, resumption of sexual intercourse, contraception, future pregnancy plans, advice about exercise, diet and weight loss, and reminders about cervical screening. There was a strong sense that GPs perceived the routine postnatal check as holistic, with references to building a relationship with the woman's wider family(32, 44, 49) and preventative medicine:(37, 45, 46, 49)

> *"The GPs interviewed did not see their role limited to routine checks and managing illness. Instead they thought their responsibility extended to assessing how the family was functioning and providing anticipatory guidance and education when appropriate."*(32)

GPs' views on when a routine postnatal check should happen, and whether there should be more than one planned contact, were not commonly discussed. Two studies made specific reference to this(37, 44) without consensus regarding the optimal time; the earliest time discussed was a review within two weeks after birth(32) and no studies described a recommendation later than 8 weeks. There was also a suggestion that the timing was woman-dependent and that 6 weeks may be right for some, but not all women. Similarly, the duration of the appointment was variable depending on the needs and complexity of the woman.

> *"six of the GPs felt that it [6 weeks] was potentially too late for young mothers: "I guess you*

For peer review only - http://bmjopen.bmj.com/site/about/guidelines.xhtml

could argue it's potentially a bit late for some women. I can certainly think of one or two quite young women who have got pregnant very quickly after having the first baby, and you wonder whether you might in some cases miss it." [GP11]"(42)

Influences on GPs' approach to postnatal care:

1. Psychological capability

Psychological capability encompasses an individual's mental functioning, including understanding and memory.(52) A broad range of knowledge was found to be required to provide postnatal care which could be explained by the variety of clinical topics included, but one study focusing on postpartum care in general found good self-reported knowledge of a basic postpartum review:

"Most PCPs [primary care physicians] *claimed to know the basic clinical examination of a postpartum woman. They were aware on the assessment of lochia cessation, wound recovery, pain control, screening for post-natal depression, breastfeeding, and preventive care, such as cervical cancer screening and contraception advice."*(46)

This was accompanied by acknowledgement that having a good basic knowledge as a generalist meant it was sometimes difficult to be expert in discrete areas:

"Participants acknowledged that the difficulty for GPs is that they offer a generalist service making it difficult for them to have expertise in all areas including PMH [perinatal mental health]*: 'You are kind of jack-of-all-trades, you are master of none'"*(44)

There was overlap between psychological capability and physical opportunity in that GPs often referred to their use or awareness of resources such as guidelines when addressing the issue of their knowledge. Recall and interpretation of guidelines was sometimes poor.

"…you're not really sure what the responsibility is or what the frequency with which you should be checking it [blood pressure] *is, and I don't think that the NICE guidance is so clear on that. (Participant 11—General Practitioner)"*(31)

Some knowledge gaps were identified in studies which explored specific topics, for example awareness of the condition itself(47), how a condition impacts the woman's mental health(48) and future risks.(50) These findings across different conditions demonstrated the vast spectrum of pathology that GPs may come across postnatally and highlights that knowledge of conditions was not universal.

"Some HCPs [healthcare professionals] *were not aware of PNA* [perinatal anxiety] *as a*

For peer review only - http://bmjopen.bmj.com/site/about/guidelines.xhtml

For peer review only

specific diagnosis and were uncertain that PNA existed as a distinct clinical entity: 'I mean it's a concept that I'm really not that much aware of either professionally or from reading.'"(47)

The knowledge required to fulfil the broad remit of postnatal care was extensive and GPs expressed they had a good basic grasp of core topics but sometimes lacked knowledge of specific clinical areas, including difficulty understanding, accessing, or interpreting guidelines.

2. Physical opportunity

This theme describes environmental and physical resources and context with reference to the action or behaviour in question(52) and was represented by all studies.(31-50) Issues such as length of time available, guidelines and screening tools, and information supplied were frequently discussed. Some elements that were representative of both physical and social opportunity are summarised here.

GPs relied on information received from other services (usually hospital) about antenatal and intrapartum journeys. Information was sometimes inadequate, whether about a specific complication or care in general. There was a desire to have improved information, with frustration expressed by words such as 'disappointing', and descriptions of difficulty accessing relevant information quickly.

"GPs also identified the need for clarity and explicit recommendations for postnatal follow-up, indicating that the process and communication could be improved: "I would be keen to do a little bit more if it was simple and clear." [GP9]"(41)

Guidelines, toolkits and screening tools were cited as resources known to or used by GPs as part of their clinical practice or continuing professional development. There was criticism of the clarity, accessibility and usability of guidelines across the different clinical topics which were out of the control of individual GPs.

"Non-availability of easily accessible, evidence based, up to date information on medicines in breastfeeding was mentioned. GPs often mentioned that their sources of information were conflicting and often "over cautious"."(38)

Exposure to perinatal learning experiences and training were described as variable, in terms of how much training GPs had overall, and that some clinical topics such as breastfeeding were not as well covered as others.

"Participants recalled limited information related to PMH [perinatal mental health] *as the focus of their specialist training was on obstetric and medical topics and in terms of practical experience: 'It can be luck of the draw what you do get exposed to' (P1)."*(44)

For peer review only - http://bmjopen.bmj.com/site/about/guidelines.xhtml

There was no clear or consistent view on how long was allocated or needed for a postnatal check. The complexity of the woman impacted on how long the appointment would take, and therefore whether all aspects of the check were afforded adequate time. Moreover, there was sometimes a tendency to avoid certain clinical topics or tasks due to a lack of time, or fear of something time-consuming emerging. GPs would sometimes arrange a follow-up appointment if not everything could initially be covered.

> *"Most felt that a more realistic time per consult should last from 15 to 20 min, compared to the current 5 to 10 min in real-time clinical practice. The limited time for consult often reduced the comprehensiveness and scope of postpartum issues covered within the postpartum visit. Consequently, the PCPs* [primary care physicians] *tended to avoid time-consuming tasks such as mental health assessment or discussing contraception options with the mothers."*(46)

In UK studies in particular, there was frustration with organisational changes meaning that health visitors and GPs were no longer co-located, impacting negatively on communication. There were positive descriptions of examples of good care when services were organised as well run multi-disciplinary teams. GPs sometimes felt disconnected by their reduced role in antenatal care which impacted on their ability to provide continuity. Organisational changes around GP and health visitor working were described as reducing opportunities for long-term relationships. However, GPs reflected positively on their professional autonomy to offer follow-up appointments when indicated. There was a recognition of the difficulties that women could face in accessing primary care services.

> *"practitioners reported that ongoing NHS reform had resulted in health visitors being moved out of general practice and into centralized services (often in non-NHS settings), a development universally regarded as deleterious to multiagency team work and delivery of effective perinatal mental healthcare"*(36)

Overall, many aspects of physical opportunity were consistently cited as important to GPs. The wide range of elements identified reflected the complexities of postnatal care itself and the intricacies of service organisation. That GPs sometimes had little or no influence over these factors impacted on their approach to patients with a sense that they wanted more time to be made available for the women with more complex issues, and better resources to complement their clinical experience.

3. Social Opportunity

Six descriptive themes were relevant to social opportunity, which encompasses aspects of social

For peer review only - http://bmjopen.bmj.com/site/about/guidelines.xhtml

norms and culture involving interactions between different people.(52) There was sometimes uncertainty from GPs about who was responsible for setting the agenda in the postnatal check or ensuring appropriate follow-up. Some GPs described a flexible approach, covering both the woman's and the GP's agenda.

> *""You've got to be quite fluid....What is it that they've come to see you about? I've got to meet that need primarily. I've got to discharge my need which is keeping them safe and running through all the possible scenarios of something going terribly wrong... anything extra that I can plug in terms of education, that's a bonus." (RGP7)"*(32)

Also influencing the nature of the consultation, was GPs citing the baby as a distraction, impacting on their care of the woman.

> *"The GPs reported that after delivery, the primary focus of care shifts from the woman with GDM to the baby."*(50)

GPs expressed a desire to understand the cultural and social background of women and how this might impact care they needed. Additionally, GPs sometimes contextualised potential barriers to compliance with care or recommendations within their perception of social norms about women finding it difficult to engage with their health and future risk.

> *"The attitudes of a mother to self-care and health were seen as a barrier. Denial of risk was perceived by some GPs to further hinder follow-up care."*(45)

Overlapping with organisational factors captured in the physical opportunity theme were GPs' perceptions of their own role, and roles of colleagues such as health visitors and midwives. GPs' minimal or absent involvement with women antenatally was cited as a reason for difficulties with postnatal care, or justification for some aspects of postnatal care being handled by someone else.

> *"A few GPs also referred in qualitative comments to their increasing distance from antenatal care due to organisational changes placing some midwives and health visitors outside practice surgeries: "I feel my role has been marginalised since joint working with health visitors has effectively stopped." (GP, survey respondent)"*(39)

This theme captured tensions GPs experienced when trying to balance conflicting consultation agendas, distractions, and evolving professional roles with their own beliefs about what women want or need postnatally.

For peer review only - http://bmjopen.bmj.com/site/about/guidelines.xhtml

4. Motivation

The COM-B Model distinguishes between reflective and automatic motivation(52), but in the analysis, motivational features were difficult to categorise as solely one of these (COM-B acknowledges that, for example, automatic processes can arise from reflection(53)) and therefore Motivation was combined as a single theme. The descriptive themes that mapped to this were 'Doctor-related factors' and 'Clinical decision making'. This theme captured GPs' perceptions about how they reached decisions, and insights into past professional or personal experiences that impacted their actions. Studies with a focus on mental health captured examples of reticence to arrive at a diagnosis, a desire to normalise aspects of perinatal mental illness, including dismissing symptoms as being part of normal life.(33-35, 39, 43)

> "[with reference to a case vignette of a traumatic birth] *"Without taking anything away from the trauma and distress suffered, there is a chance that this also could be something this woman could work through and doesn't necessarily see a doctor about and manages to recover from quite well without needing medical intervention." GP 6 (Male)"*(43)

GPs sometimes resisted their instinct or known expectation to explore perinatal mental health issues for reasons such as desire to avoid awkwardness.

> *"'Can I be honest with you sometimes I wonder if you really want to open this can of worms and it's so much easier just to jolly along and check the BP, check the urine, check this and that and have them out the door and see the next patient' (P5)."*(44)

In some cases, social opportunity seemed to impact on motivation: the nature of relationships between healthcare professions or between GPs and women influenced the motivation of the GP to act in a certain way. For example, when continuity of care was not achieved, GPs were less motivated to be thorough:

> *"Some health professionals described consciously inhibiting disclosure in order not to be placed in this position citing lack of continuity of care as the reason: "Easier not to ask, if I'm not going to see her again." (L GP1)"*(34)

Some factors appeared to increase GPs' confidence in making a diagnosis or enabling disclosure of symptoms such as involvement of others in decision making, use of clinical instinct, and patient insight into their symptoms.

> *"'Your antenna would be raised by people coming clean with you that there is something*

For peer review only - http://bmjopen.bmj.com/site/about/guidelines.xhtml

going on…a history… the usual kind of joy isn't there, they're, quiet' (P5)."(44)

GPs' views about their roles most commonly fell within the social opportunity theme, but there were motivational influences whereby beliefs about their role could impact the nature of their interaction with the woman.

"Many considered their main purpose as treating physical health problems and not to promote health."(49)

Overall, there was a reliance on experience and clinical acumen and a desire to share decision making with the woman and professional colleagues. This was combined with some resistance to completing a diagnostic process when pathology had been identified or even avoiding the diagnostic process altogether.

DISCUSSION

This review suggested wide ranging influences in GPs' overall approach to postnatal care, the strongest being physical and social opportunity, and that these issues impact on motivational factors. GPs viewed the task of providing care for women postnatally as more challenging when they lacked time, clear clinical guidance, and access to suitable communication from secondary care. GPs derived professional satisfaction and enjoyment in seeing women postnatally, as part of their care for the whole family but were reluctant to provide a comprehensive postnatal check when constrained by time or perceived a risk of awkwardness. It was perhaps surprising that very little was found regarding the timing of a routine postnatal check, given its lack of evidence base. There was uncertainty about whether 6-8 weeks was the ideal time, but also an unquestioning acceptance that 6-8 weeks was the norm. This could be explained by 6-8 weeks being a cultural norm, being recommended in guidelines and coinciding with the infant physical examination and first immunisations. The absence of research to determine the optimal time for a routine postnatal check could explain why GPs lack a clear sense of what might be ideal, and usual practice continuing without formal evaluation.

Limitations of this review included the heterogeneity between studies which was anticipated but resulted in the synthesis of data from studies about different discrete clinical topics. This was considered throughout the analysis, and findings from studies concerning postnatal care as a broad topic contained similar findings, giving some assurance to this approach. The inclusion of non-GP participants in some studies meant that during data extraction, it was sometimes difficult to be certain which data was from GPs. Although there are examples globally of similar postnatal care models to the UK, there are important differences that could influence the analysis, for example, studies from Australia and Singapore reference different needs of private and public patients which is not so

For peer review only - http://bmjopen.bmj.com/site/about/guidelines.xhtml

relevant to the UK. Finally, whilst the included studies were all appraised against CASP criteria, one was a report not formally peer-reviewed.(39)

Utilisation of the COM-B model as part of the analysis was made after the team reflected that this model was able to neatly frame what the data were showing. Application of the COM-B model to the synthesis allowed for translation of findings to the model's associated Behaviour Change Wheel (BCW)(52) and therefore the generation of potential intervention or policy areas that apply to each behaviour. The strongest influences of social and physical opportunity revealed intervention categories of education, persuasion, incentivisation and coercion. GP practices have already been financially incentivised to offer women a postnatal check since the 2020 change to the NHS England GP contract, but this has not been accompanied by complementary interventions such as education or persuasion. It may be possible in the future to determine whether this intervention has improved postnatal check provision, but it will not measure quality. By targeting interventions recommended in the BCW from the motivation and social opportunity themes (such as enablement, training, education), quality and consistency could be improved.

The postnatal period is one of marked health inequality.(7) This is a critical issue that requires further research to better understand causes, and intervention to overcome it. This review found minimal reference to differential risk between populations, except for one study which looked specifically at Black and ethnic minority women,(36) and higher risk groups were not well defined or explained in included studies. The suggestion within the physical opportunity theme that different women needed different lengths of time for a postnatal review may be important in addressing this inequality; if women at higher risk of morbidity or mortality could be identified, they could be given a longer appointment, or additional follow-up. From what is already know about disparities in outcomes for women with different social and medical backgrounds, it may be reasonable to extrapolate that adjusting routine care to meet needs has the potential to improve outcomes and reduce inequality.

Future research should address the issue of timing of the routine maternal postnatal check, both when it should happen, and how long it should take. These research areas should be approached with specific reference to groups known to be most at risk. In the UK context, the introduction of a mandatory maternal postnatal check to the GP contract should be evaluated, from the perspectives of women, but also GPs who provide it, so that its provision and quality are optimised.

For peer review only - http://bmjopen.bmj.com/site/about/guidelines.xhtml

Figure Legends

Figure 1: Study selection process

Figure 2: The Behaviour Change Wheel(52)

Figure 3: Themes mapped to Com-B

Contributorship statement

All authors contributed to the design, study selection, analysis and writing and approved the final manuscript.

Competing interests

There are no competing interests for any author.

Funding

Clare Macdonald is funded by the National Institute for Health Research (NIHR) West Midlands Applied Research Collaboration. The views expressed are those of the author and not necessarily those of the NIHR or the UK Department of Health and Social Care.

Data sharing statement

There are no plans to store further data in an open repository but much of the review data has been submitted in supplementary files and the corresponding author can be contacted if there are any future requests for further data.

Ethics approval statement

This is a qualitative systematic review and ethics approval was not required. The ethical considerations and permissions in the studies included in the review were considered as part of the quality appraisal process.

Patient and Public Involvement

There was no specific public and patient involvement in this systematic review.

For peer review only - http://bmjopen.bmj.com/site/about/guidelines.xhtml

REFERENCES

1. The National Health Service (General Medical Services Contracts) Regulations 2015 [Available from: https://www.legislation.gov.uk/uksi/2015/1862/regulation/3. Accessed 10/2022
2. NHS England BMA. Investment and Evolution - Update to the GP Contract Agreement 2021-23/24 2020 [Available from: https://www.england.nhs.uk/publication/investment-and-evolution-update-to-the-gp-contract-agreement-20-21-23-24/. Accessed 10/2022
3. The National Federation of Women's Institutes N. Support overdue: Women's experiences of maternity services. 2017 [Available from: https://www.thewi.org.uk/__data/assets/pdf_file/0009/187965/NCT-nct-WI-report-72dpi.pdf. Accessed 10/2022
4. Cumberlege J. Better Births. Improving outcomes of maternity services in England. A Five Year Forward. View for maternity care. 2016 [Available from: https://www.england.nhs.uk/wp-content/uploads/2016/02/national-maternity-review-report.pdf. Accessed 10/2022
5. Public Health England. Breastfeeding prevalence at 6 to 8 weeks after birth: Annual data statistical commentary 2019 to 2020 2021 [Available from: https://assets.publishing.service.gov.uk/government/uploads/system/uploads/attachment_data/file/957253/OFF_SEN_Annual_Breastfeeding_Statistical_Commentary_2019_2020.pdf. Accessed 10/2022
6. NICE. National Institute for Health and Care Excellence: Postnatal Care guideline (NG194) 2021 [Available from: https://www.nice.org.uk/guidance/ng194. Accessed 10/2022
7. MBRRACE-UK. Saving Lives, Improving Mothers' Care. Lessons learned to inform maternity care from the UK and Ireland Confidential Enquiries into Maternal Deaths and Morbidity 2016-18 2020 [Available from: https://www.npeu.ox.ac.uk/assets/downloads/mbrrace-uk/reports/maternal-report-2020/MBRRACE-UK_Maternal_Report_Dec_2020_v10_ONLINE_VERSION_1404.pdf. Accessed 10/2022
8. Haran C, van Driel M, Mitchell BL, et al. Clinical guidelines for postpartum women and infants in primary care-a systematic review. *BMC Pregnancy Childbirth.* 2014;14:51.
9. Public Health Agency of Canada. Family-centred maternity and newborn care: National guidelines 2021 [Available from: https://www.canada.ca/en/public-health/services/maternity-newborn-care-guidelines.html. Accessed 10/2022
10. Royal Austrailian College of General Practitioners. Maternity Care in General Practice. Position. 2021 [21.6.21]. Available from: https://www.racgp.org.au/advocacy/position-statements/view-all-position-statements/clinical-and-practice-management/maternity-care-in-general-practice#ref-num-10. Accessed 10/2022
11. Smith A. Shakespeare J. Dixon A. The role of GPs in maternity care – what does the future hold? : The King's Fund; 2010.
12. NICE. National Institute for Health and Care Excellence: Antenatal care guideline (NG201) 2021 [Available from: https://www.nice.org.uk/guidance/ng201. Accessed 10/2022
13. The National Health Service (General Medical Services Contracts) Regulations 2004 No. 291 [Available from: https://www.legislation.gov.uk/uksi/2004/291/contents/made. Accessed 10/2022
14. NCT. The Hidden Half: Bringing postnatal mental illness out of hiding 2017 [Available from: https://www.nct.org.uk/sites/default/files/related_documents/739-NCT-theHiddenHalf-report-150dpi_0.pdf. Accessed 10/2022
15. RCGP. GPs doing very best for new mothers but need more time and resources, says RCGP 2017 [Available from: https://www.rcgp.org.uk/about-us/news/2017/june/gps-doing-very-best-for-new-mothers-but-need-more-time-and-resources.aspx. Accessed 10/2022
16. Lithgow GE, Rossi J, Griffin SJ, et al. Barriers to postpartum diabetes screening: a qualitative synthesis of clinicians' views. *British Journal of General Practice*. 2021;71(707):E473-E82.

For peer review only - http://bmjopen.bmj.com/site/about/guidelines.xhtml

17. Makama M, Awoke MA, Skouteris H, et al. Barriers and facilitators to a healthy lifestyle in postpartum women: A systematic review of qualitative and quantitative studies in postpartum women and healthcare providers. *Obesity Reviews*. 2021;22(4):e13167.
18. Munabi-Babigumira S, Lewin S, Glenton C, et al. Factors that influence the provision of postnatal care by health workers: a qualitative evidence synthesis. *Cochrane Database of Systematic Reviews*. 2021(7).
19. NCT. NCT finds a quarter of new mothers are not asked about their mental health 2021 [Available from: https://www.nct.org.uk/about-us/media/news/nct-finds-quarter-new-mothers-are-not-asked-about-their-mental-health. Accessed 10/2022
20. Booth A, Noyes J, Flemming K et al. Formulating questions to explore complex interventions within qualitative evidence synthesis. *BMJ Global Health*. 2019;4(Suppl 1):e001107.
21. OECD. DAC List of ODA Recipients 2021 [Available from: https://www.oecd.org/dac/financing-sustainable-development/development-finance-standards/daclist.htm. Accessed 10/2022
22. Thomas J, Harden A. Methods for the thematic synthesis of qualitative research in systematic reviews. *BMC Medical Research Methodology*. 2008;8(1):45.
23. Noonan M, Doody O, Jomeen J,et al. Family physicians perceived role in perinatal mental health: an integrative review. *BMC Family Practice*. 2018;19(1):154.
24. Ibanez G, Zabar J, Cadwallader JS, et al. Views of general practitioners on indoor environmental health risks in the perinatal period. *Frontiers in Medicine*. 2015;2:32.
25. Weingarten MA, Reinitz A, Hart J. Attitudes to primary-care gynaecology among family physicians and gynaecologists in Israel. *Scandinavian Journal of Primary Health Care*. 1992;10(1):36-41.
26. Naughton F, Hopewell S, Sinclair L, et al. Barriers and facilitators to smoking cessation in pregnancy and in the post-partum period: The health care professionals' perspective. *British journal of health psychology*. 2018;23(3):741-57.
27. Kline CR, Martin DP, Deyo RA. Health consequences of pregnancy and childbirth as perceived by women and clinicians. *Obstetrics & Gynecology*. 1998;92(5):842-8.
28. Green A, Callaway L, McIntyre HD, et al. Diagnosing and providing initial management for patients with Gestational Diabetes: What is the General Practitioner's experience? *Diabetes Research & Clinical Practice*. 2020;166:108290.
29. Rivas K, Schmuhl NB, Taboada M, et al. Are Wisconsin resident physicians trained in immediate postpartum long acting reversible contraception? *Contraception.* 2021;104(4):372-6.
30. Kean LJ, Hamilton J, Shah P. Antidepressants for mothers: what are we prescribing? *Scottish Medical Journal*. 2011;56(2):94-7.
31. Bick D, Silverio SA, Bye A, et al. Postnatal care following hypertensive disorders of pregnancy: a qualitative study of views and experiences of primary and secondary care clinicians. *BMJ Open*. 2020;10(1):e034382.
32. Brodribb W, Zadoroznyj M, Dane A. The views of mothers and GPs about postpartum care in Australian general practice. *BMC Family Practice*. 2013;14:139.
33. Chew-Graham C, Chamberlain E, Turner K, et al. GPs' and health visitors' views on the diagnosis and management of postnatal depression: a qualitative study. *British Journal of General Practice*. 2008;58(548):169-76.
34. Chew-Graham CA, Sharp D, Chamberlain E, et al. Disclosure of symptoms of postnatal depression, the perspectives of health professionals and women: a qualitative study. *BMC Family Practice*. 2009;10(1):7.
35. Dijkhuis T, Bloem F, Kusters L, et al. Investigating the current knowledge and needs concerning a follow-up for long-term cardiovascular risks in Dutch women with a preeclampsia history: a qualitative study. *BMC Pregnancy and Childbirth*. 2020;20.
36. Edge D. Falling through the net - black and minority ethnic women and perinatal mental healthcare: health professionals' views. *Gen Hosp Psychiatry*. 2010;32(1):17-25.

For peer review only

For peer review only - http://bmjopen.bmj.com/site/about/guidelines.xhtml

37. Hewage SS, Singh SR, Chi C, et al. Health Care Providers' Perceptions of Responsibilities and Resources to Reduce Type 2 Diabetes Risk After Gestational Diabetes Mellitus. *Clin Diabetes.* 2018;36(2):160-7.
38. Jayawickrama HS, Amir LH, Pirotta MV. GPs' decision-making when prescribing medicines for breastfeeding women: Content analysis of a survey. *BMC Research Notes.* 2010;3:82.
39. Khan L. Falling through the gaps: perinatal mental health and general practice. www.centreformentalhealth.org.uk; 2015.
40. Kilgour C, Bogossian FE, Callaway L, et al. Postnatal gestational diabetes mellitus follow-up: Perspectives of Australian hospital clinicians and general practitioners. *Women & Birth: Journal of the Australian College of Midwives*. 2019;32(1):e24-e33.
41. Kilgour C, Bogossian F, Callaway L, et al. Experiences of women, hospital clinicians and general practitioners with gestational diabetes mellitus postnatal follow-up: A mixed methods approach. *Diabetes Research & Clinical Practice.* 2019;148:32-42.
42. Lunniss H, Cameron S, Chen ZE. Views of general practitioners on providing contraceptive advice and long-acting reversible contraception at the 6-week postnatal visit: a qualitative study. *Journal of Family Planning and Reproductive Health Care*. 2016;42(2):99-105.
43. Mortimer H, Habash-Bailey H, Cooper M, et al. An exploratory qualitative study exploring GPs' and psychiatrists' perceptions of post-traumatic stress disorder in postnatal women using a fictional case vignette. *Stress and Health*. 2021;38(3):544-555.
44. Noonan M, Doody O, O'Regan A, et al Irish general practitioners' view of perinatal mental health in general practice: a qualitative study. *BMC Family Practice*. 2018;19(1):196.
45. Pennington AVR, O'Reilly SL, Doris Y, et al. Improving follow-up care for women with a history of gestational diabetes: perspectives of GPs and patients. *Australian Journal of Primary Health*. 2017;23(1):66-74.
46. Poon Z, Lee ECW, Ang L, et al. Experiences of primary care physicians managing postpartum care: a qualitative research study. *BMC Family Practice*. 2021;22(1).
47. Silverwood V, Nash A, Chew-Graham CA, et al. Healthcare professionals' perspectives on identifying and managing perinatal anxiety: a qualitative study. *British Journal of General Practice.* 2019;69(688):E768-E76.
48. Sriranjan AN, Abrams R, Wong G, et al. Towards an understanding of GPs' viewpoint on diagnosing postnatal depression in general practice: a small-scale realist evaluation. *Primary Health Care Research and Development.* 2020;21.
49. Talbot H, Strong E, Peters S, et al. Behaviour change opportunities at mother and baby checks in primary care: a qualitative investigation of the experiences of GPs. *British Journal of General Practice*. 2018;68(669):e252-e9.
50. Timm A, Nielsen KK, Christensen U, et al. Healthcare Professionals' Perspectives on the Cross-Sectoral Treatment Pathway for Women with Gestational Diabetes during and after Pregnancy-A Qualitative Study. *Journal of Clinical Medicine*. 2021;10(4).
51. Critical Appraisal Skills Programme. CASP qualitative checklist 2019 [23.06.2021]. Available from: https://casp-uk.net/casp-tools-checklists/. Accessed 10/2022
52. Michie S, van Stralen MM, West R. The behaviour change wheel: A new method for characterising and designing behaviour change interventions. *Implementation Science*. 2011;6(1):42.
53. Michie S, West R. Behaviour change theory and evidence: a presentation to Government. *Health Psychology Review*. 2013;7(1):1-22.

For peer review only - http://bmjopen.bmj.com/site/about/guidelines.xhtml

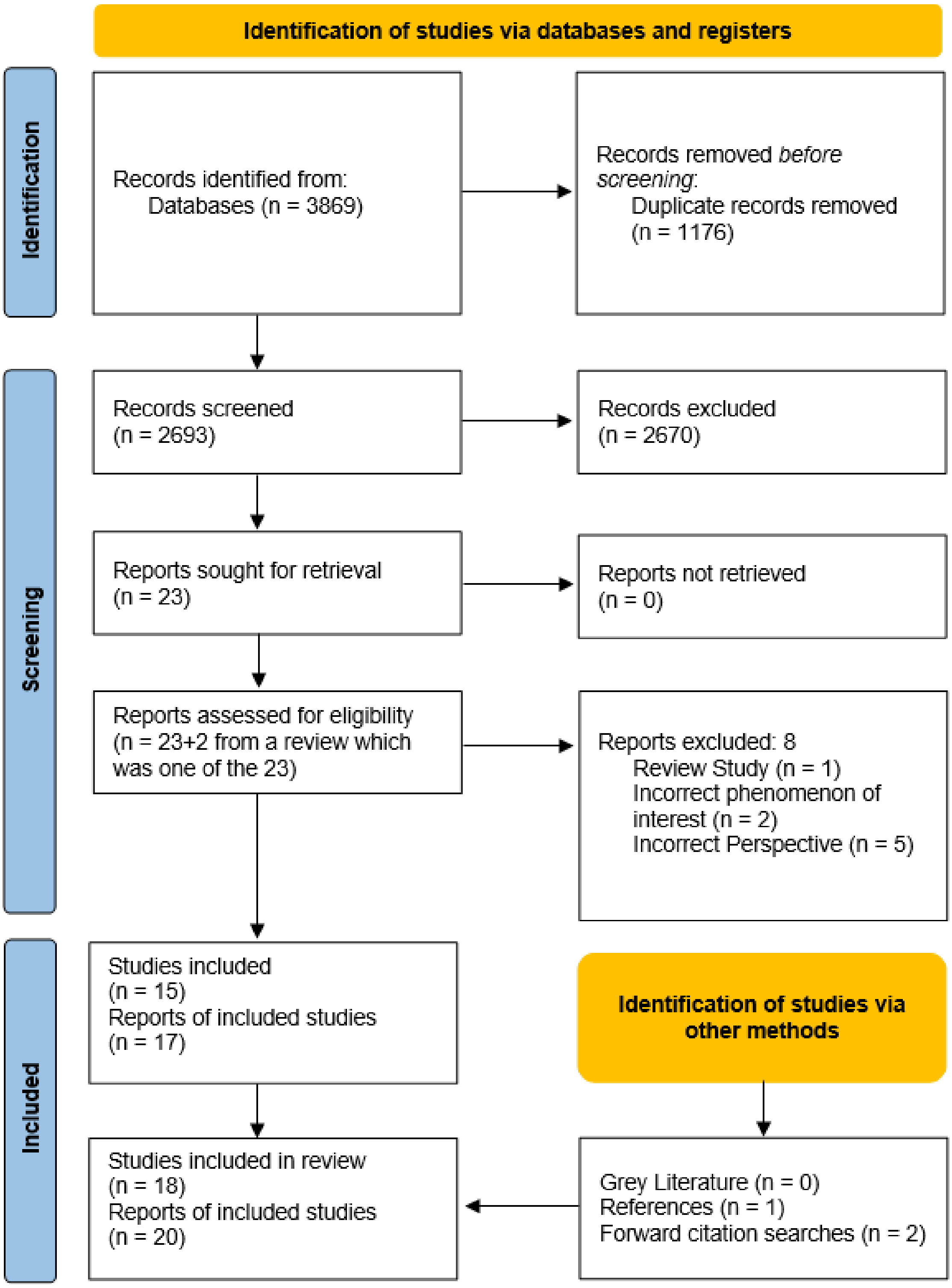
Identification of studies via databases and registers
Identification
Records identified from:
Databases (n = 3869)
Records removed *before screening*:
Duplicate records removed (n = 1176)
Screening
Records screened
(n = 2693)
Records excluded
(n = 2670)
Reports sought for retrieval
(n = 23)
Reports not retrieved
(n = 0)
Reports assessed for eligibility
(n = 23+2 from a review which was one of the 23)
Reports excluded: 8
Review Study (n = 1)
Incorrect phenomenon of interest (n = 2)
Incorrect Perspective (n = 5)
Included
Studies included
(n = 15)
Reports of included studies
(n = 17)
Identification of studies via other methods
Studies included in review
(n = 18)
Reports of included studies
(n = 20)
Grey Literature (n = 0)
References (n = 1)
Forward citation searches (n = 2)

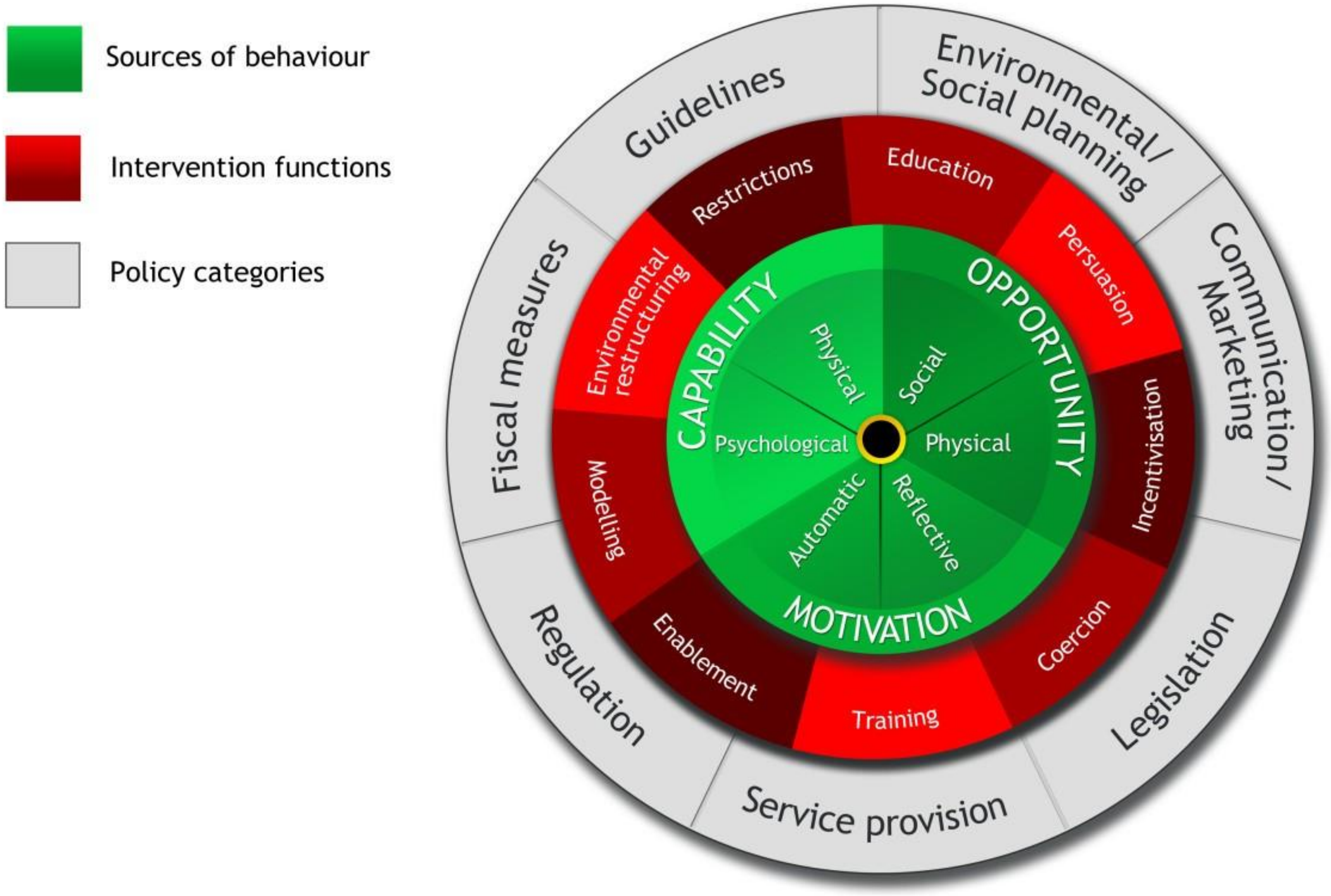
Sources of behaviour
Intervention functions
Policy categories
Guidelines
Environmental/ Social planning
Communication/ Marketing
Legislation
Service provision
Regulation
Fiscal measures
Restrictions
Education
Persuasion
Incentivisation
Coercion
Training
Enablement
Modelling
Environmental restructuring
CAPABILITY
OPPORTUNITY
MOTIVATION
Physical
Psychological
Social
Physical
Automatic
Reflective

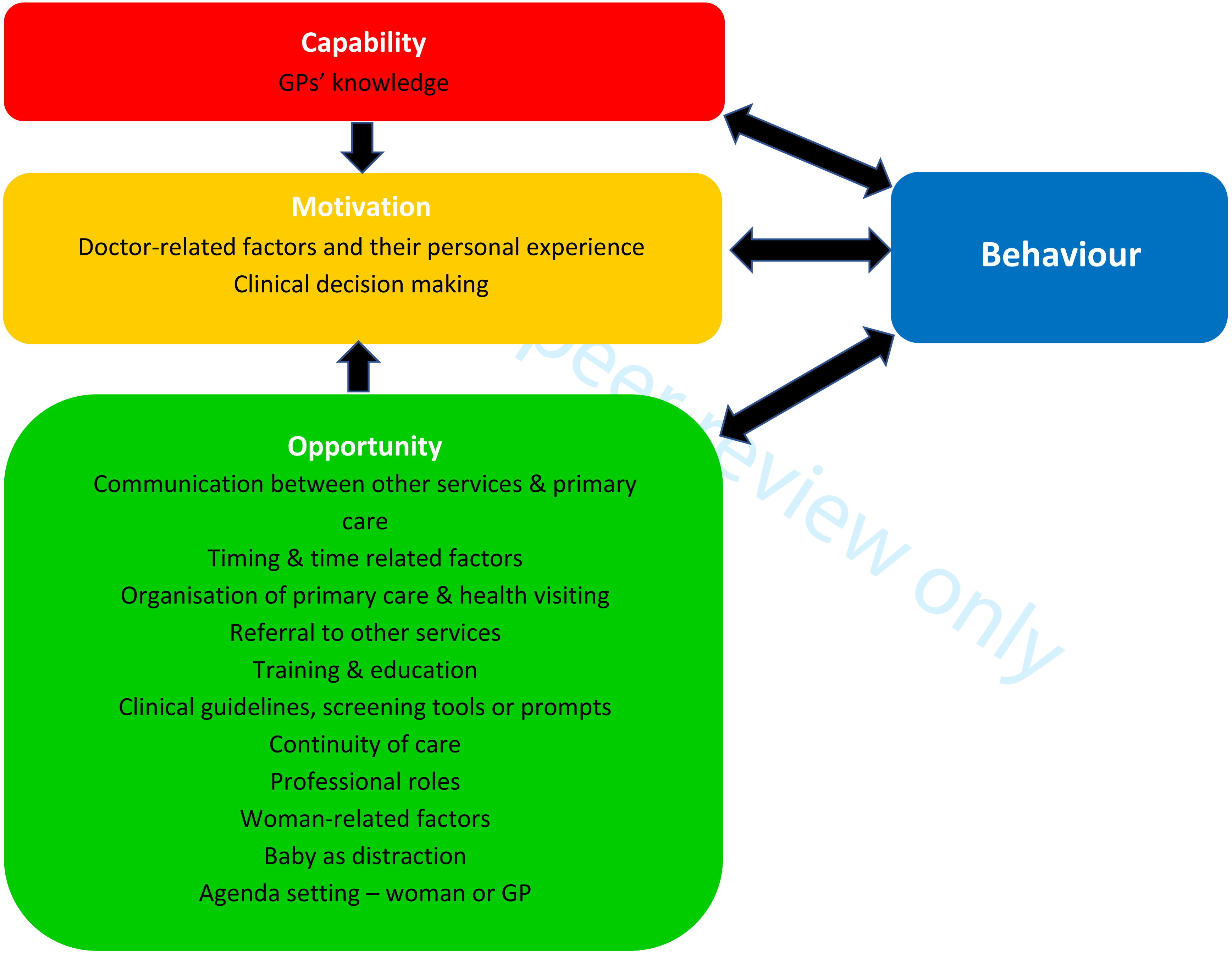

For peer review only - http://bmjopen.bmj.com/site/about/guidelines.xhtml

Supplementary File 1: Search Terms

OR<br>↓		OR<br>↓		OR<br>↓
MeSH Terms:<br>General Practitioners/<br>Primary Health Care/<br>Physicians, Primary Care/<br>Physicians, Family/<br><br>‘general practitioner*’,<br>‘GP*’	**AND**	MeSH Terms:<br>Perinatal Care/<br>Postpartum Period/<br>Postnatal Care/<br>Maternal Health/<br>Puerperal Disorders exp/<br>Diabetes, Gestational/<br>Hypertension, Pregnancy Induced exp/<br>Urinary Incontinence exp/<br>Pelvic Organ Prolapse/<br><br>‘perinatal’<br>‘postpartum’<br>‘postnatal’	**AND**	MeSH Terms:<br>Attitude/<br>Attitude of health personnel/<br>Perception/<br><br>‘attitude*’<br>‘belief*’<br>‘opinion*’<br>‘understanding*’<br>‘viewpoint*’<br>‘perspective*’<br>‘perception*’<br>‘view*’<br>‘experience*’

Summary of study characteristics

Title	Author	Year	Setting	Recruitment	Number of GPs	Additional participants	Focus of study	Data collection from GPs	Analysis methodology	Comments
**Postnatal care following hypertensive disorders of pregnancy: a qualitative study of views and experiences of primary and secondary clinicians**	Bick et al	2019	UK	GPs invited from practices in 3 London maternity unit areas	5	Obstetricians, physician, health visitors, midwives (n=15)	Views of clinicians caring for postnatal women who had HDP	Semi-structured interviews	Thematic	
**The views of mothers and GPs about postpartum care in Australian general practice**	Brodribb et al	2013	Australia	GPs in locations serving hospitals taking part in a postnatal care study	6	Mothers (n=88)	Views about postpartum care in general practice	Interviews	Thematic	Reports on its pilot, and larger study together. Only one theme reported in full, authors contacted but no response
**GPs' and health visitors' views on the diagnosis and management of postnatal depression**	Chew-Graham et al	2008	UK	Invitation via practices who were participating in an associated RCT. Purposive by GP demographics.	19	Health visitors (n=14)	Views on role in detection and management of postnatal depression	In-depth interviews	Thematic	
**Disclosure of symptoms of postnatal depression, the perspectives of health professionals and women: a qualitative study**	Chew-Graham et al	Reports on same study as above								
**Investigating the current knowledge and needs concerning follow-up for long-term cardiovascular risks in Dutch women with a preeclampsia history: a qualitative study**	Dijkhuis et al	2020	Netherlands	Convenience, GP offices in region of hospital study base	4	Women, obstetricians, vascular medicine specialists, cardiologists (n=25)	Opinions and wishes about follow-up for women with a history of pre-ecplampsia	Semi-structured interviews	Thematic	
**Falling through the net - Black and minority ethnic women and perinatal mental healthcare: health professionals' views**	Edge	2010	UK	GPs from areas with high Black Caribbean population	5	Midwives, hospital doctors, health visitors (n=37)	Investigating views about perinatal mental healthcare for BAME women	In-depth interviews	Framework	
**Health Care Providers' Perceptions of Responsibilities and Resources to Reduce Type 2 Diabetes Risk After Gestational Diabetes Mellitus**	Hewage et al	2018	Singapore	Purposive, snowball sampling	5	OB/GYN, endocrinologists, dietitians, nurses (n=27)	Healthcare providers' perceptions of care responsibilities and resources related to reducing T2DM among women with previous GDM	In-depth interviews	Framework	
**GPs' decision-making when prescribing medicines for**	Jayawickrama et al	2010	Australia	Anonymous postal questionnaire	253	-	Factors influencing GPs' decision-making when	Survey	Thematic Networks	

**breastfeeding women: Content analysis of a survey**							prescribing for breastfeeding women			
**Falling through the gaps: perinatal mental health and general practice**	Khan	2018	UK	Convenience (large scale dissemination through virtual portals)	43	Women (n=1551)	Experiences of interactions between women with common perinatal mental health problems and GPs	Survey and semi-structured interviews	Interpretative phenomenological approaches	
**Postnatal gestational diabetes mellitus follow-up: Perspectives of Australian hospital clinicians and general practitioners**	Kilgour et al	2018	Australia	GPs who worked in collaboration with a large city hospital (where the hospital clinicians were recruited from)	16	Hospital clinicians (n=13)	Communication processes between GPs and hospital clinicians providing postnatal GDM care		Computer automated Leximancer analysis	
**Experiences of women, hospital clinicians and general practitioners with gestational diabetes mellitus follow-up: A mixed methods approach**	Kilgour et al	Reports on same study as above, but with 2 further (quant) stages. Some additional GP qual data included in this publication to be included.								
**Views of general practitioners on providing contraceptive advice and long-acting eversible contraception at the 6-week postnatal visit: a qualitative study**	Lunniss et al	2015	UK	Purposive (practice demographics), , convenience (education event), snowball	13	-	Views on contraception provision including LARCs at 6 week check	Semi-structured interviews	Thematic	
**An exploratory qualitative study exploring GPs' and psychiatrists' perceptions of post-traumatic stress disorder in postnatal women using a fictional case vignette**	Mortimer et al	2021	UK	Email invitation / poster adverts (not explicit whether purposive)	6	Psychiatrists (n=7)	GP and psychiatrist perceptions of PTSD in postnatal women	Semi-structured interviews with a case vignette	Framework	
**Irish general practitioners' view of perinatal mental health in general practice: a qualitative study**	Noonan et al	2018	Ireland	Purposive, GPs connected with GP tutors from	10	-	Caring for women with perinatal mental illness	Semi-structured interviews	Thematic	
**Improving follow-up care for women with a history of gestational diabetes: perspectives of GPs and patients**	Pennington et al	2017	Australia	Purposive (GP and practice demographics)	18	Women (n=16)	Perspectives of factors influencing engagement with diabetes prevention after a pregnancy with GDM	Semi-structured interviews	Thematic content	
**Experiences of primary care physicians managing postpartum care: a qualitative research study**	Poon et al	2021	Singapore	Purposive (demographics), networks of the researchers	29	-	Managing postpartum women	Focus Groups<br>In depth interviews	?thematic, unclear	
**Healthcare professionals' perspectives on identifying and managing perinatal anxiety: a qualitative study**	Silverwood et al	2019	UK	GPs via WM-CRN and personal contacts	10	Midwives, HVs, (n=12)	Identification and management of perinatal anxiety	Semi-structured interviews	Constant comparison	

For peer review only - http://bmjopen.bmj.com/site/about/guidelines.xhtml

**Towards an understanding of GPs' viewpoint on diagnosing postnatal depression in general practice: a small-scale realist evaluation**	Sriranjan et al	2020	UK	Convenience sample, single GP practice	7	-		Semi-structured interviews with case vignettes	Framework analysis	
**Behaviour change opportunities at mother and baby checks in primary care: a qualitative investigation of the experiences of GPs**	Talbot et al	2018	UK	Snowball sampling GP practices	18	-	Health behaviour change opportunities at the postnatal check	Semi-structured interviews - phone	Thematic analysis	
**Healthcare Professionals' Perspectives on the Cross-Sectoral Treatment Pathway for Women with Gestational Diabetes during and after Pregnancy – A Qualitative Study**	Timm et al	2021	Denmark	Purposive to cover antenatal and postnatal period, by email / phone (selection not clearly stated)	2	Midwives, obstetricians, nurse, health visitors (n=15)	Management of GDM in and after pregnancy	Semi-structured interviews	Systematic Text Condensation	

For peer review only

Supplementary File 3: CASP Assessment Table

	Bick et al (2019)	*Brodribb et al (2013)*	*Chew-Graham et al (2008)*	*Dijkhuis et al (2020)*	*Edge et al (2010)*	*Hewage et al (2018)*	*Jayawickrama et al (2010)*	*Kham (2015)*	*Kilgour et al (2018*	*Lunniss et al (2015)*	*Mortimer et al (2021)*	*Noonan et al (2018)*	*Pennington et al (2017)*	*Poon et al (2021)*	*Silverwood et al (2019)*	*Sriranjan et al (2020)*	*Talbot et al (2018)*	*Timm et al (2021)*
**Was there a clear statement of the aims of the research?**	Y	Y	Y	Y	Y	Y	Y	N	Y	Y	Y	Y	Y	Y	Y	Y	Y	Y
**Is a qualitative methodology appropriate?**	Y	Y	Y	Y	Y	Y	Y	Y	Y	Y	Y	Y	Y	Y	Y	Y	Y	Y
**Was the research design appropriate to address the aims of the research?**	Y	Y	Y	Y	Y	Y	Y	Unclear	Y	Y	Y	Y	Y	Y	Y	Y	Y	Y
**Was the recruitment strategy appropriate to the aims of the research?**	Y	Y	Y	Y	Y	Y	Y	N	Y	Y	Y	Y	Y	Y	Y	Y	Y	Y
**Was the data collected in a way that addressed the research issue?**	Y	Y	Y	Y	Y	Y	N	Y	Y	Y	Y	Y	Y	Y	Y	Y	Y	Y
**Has the relationship between researcher and participants been adequately considered?**	Unclear	N	N	Y	N	N	N	N	Y	N	Y	Y	N	Y	N	Y	N	N
**Have ethical issues been taken into consideration?**	Y	Y	Y	Y	Y	Y	Y	Unclear	Y	Y	Y	Y	Y	Y	Y	Y	Y	Y
**Was the data analysis sufficiently rigorous?**	Y	Y	Y	Y	Y	Y	Y	N	Y	N	Y	Y	Y	Y	Y	Y	Y	Y
**Is there a clear statement of findings?**	Y	Y	Y	Y	Y	Y	Y	Y	Y	Y	Y	Y	Y	Y	Y	Y	Y	Y
**How valuable is the research; will the results help locally?**	Y	Y	Y	Y	Y	N	Y	Y	Y	Y	Y	Y	Y	Y	Y	Y	Y	Y

Supplementary File 4: Descriptive Themes

Descriptive Theme	Description / definition
Content and timing of postnatal checks	Any reference to content or timing of postnatal reviews, including follow-up or monitoring of pregnancy-specific pathology
Agenda setting - woman or GP	GPs' beliefs about what women want to address, or references to GPs deciding what they will cover
Baby as distraction	Baby issues taking priority, focus being baby-driven postnatally
Clinical decision making	Beliefs about diagnoses and clinical management, descriptions about how pathology is managed
Communication between other services and primary care	Communication from hospital to primary care, or other services and primary care, including notification of which patients need specific follow-up etc
Continuity of care	Specific mentions of continuity of care – either services separate from primary care (e.g. views on midwifery continuity of care) or GP views on GP continuity of care
Doctor-related factors and personal experience	Characteristics / descriptive statements about doctors that influence (positive or negative) care including personal experience
Guidelines, screening tools and prompts	Including use of guidelines, prompts or screening tools and references to their use
Knowledge	What GPs say they know, or do not know
Organisation of primary care and health visiting	GP services, including access e.g. booking appointments, arranging follow-up and descriptions about how HV is organised, including how it has changed
Referral to other services	Including accessing patient-specific advice
Role of the GP and MDT members	Descriptions of the nature of the role of the GP and other healthcare professionals e.g. HV / pharmacists / midwives
Time-related factors	Including duration of the appointment, issues that impact time pressures
Training and education	References to training received, or training wanted
Woman-related factors	GPs views on factors about women that influences care, including involvement (or not) of their partners

Mapping descriptive themes to the COM-B domains

COM-B domain	Descriptive Themes	Sources
**Psychological Capability**	Knowledge	(31, 35, 40, 41, 44, 47, 49, 50)
**Physical Capability**	None	
**Physical Opportunity**	Time-related factors<br>Screening tools or prompts<br>Organisation of primary care and health visiting<br>Referral to other services<br>Continuity of care<br>Communication between services<br>Training and education<br>Clinical guidelines, screening tools or prompts	(31-50)
**Social Opportunity**	Woman-related factors<br>Baby as distraction<br>Antenatal and intrapartum experiences<br>Agenda setting<br>GP roles<br>MDT roles	(31-42, 44-46, 48-50)
**Reflective and Automatic Motivation**	Doctor-related factors and personal experience<br>Clinical decision making	(32-34, 37, 38, 39, 43-49)

PRISMA 2020 Checklist

Section and Topic	Item #	Checklist item	Location where item is reported
**TITLE**			
Title	1	Identify the report as a systematic review.	Title p1
**ABSTRACT**			
Abstract	2	See the PRISMA 2020 for Abstracts checklist.	Abstract p2
**INTRODUCTION**			
Rationale	3	Describe the rationale for the review in the context of existing knowledge.	Background p5
Objectives	4	Provide an explicit statement of the objective(s) or question(s) the review addresses.	Aims p5
**METHODS**			
Eligibility criteria	5	Specify the inclusion and exclusion criteria for the review and how studies were grouped for the syntheses.	Methods p6-7
Information sources	6	Specify all databases, registers, websites, organisations, reference lists and other sources searched or consulted to identify studies. Specify the date when each source was last searched or consulted.	Information sources p8
Search strategy	7	Present the full search strategies for all databases, registers and websites, including any filters and limits used.	Supp file 1
Selection process	8	Specify the methods used to decide whether a study met the inclusion criteria of the review, including how many reviewers screened each record and each report retrieved, whether they worked independently, and if applicable, details of automation tools used in the process.	Screening and data extraction p8
Data collection process	9	Specify the methods used to collect data from reports, including how many reviewers collected data from each report, whether they worked independently, any processes for obtaining or confirming data from study investigators, and if applicable, details of automation tools used in the process.	Screening and data extraction p8
Data items	10a	List and define all outcomes for which data were sought. Specify whether all results that were compatible with each outcome domain in each study were sought (e.g. for all measures, time points, analyses), and if not, the methods used to decide which results to collect.	Data synthesis p8-9
	10b	List and define all other variables for which data were sought (e.g. participant and intervention characteristics, funding sources). Describe any assumptions made about any missing or unclear information.	Data synthesis p8-9
Study risk of bias assessment	11	Specify the methods used to assess risk of bias in the included studies, including details of the tool(s) used, how many reviewers assessed each study and whether they worked independently, and if applicable, details of automation tools used in the process.	Quality appraisal p9
Effect measures	12	Specify for each outcome the effect measure(s) (e.g. risk ratio, mean difference) used in the synthesis or presentation of results.	n/a
Synthesis methods	13a	Describe the processes used to decide which studies were eligible for each synthesis (e.g. tabulating the study intervention characteristics and comparing against the planned groups for each synthesis (item #5)).	n/a
	13b	Describe any methods required to prepare the data for presentation or synthesis, such as handling of missing summary statistics, or data conversions.	Data synthesis p8-9
	13c	Describe any methods used to tabulate or visually display results of individual studies and syntheses.	Data synthesis p8-9
	13d	Describe any methods used to synthesize results and provide a rationale for the choice(s). If meta-analysis was performed, describe the model(s), method(s) to identify the presence and extent of statistical heterogeneity, and software package(s) used.	Data synthesis p8-9
	13e	Describe any methods used to explore possible causes of heterogeneity among study results (e.g. subgroup analysis, meta-regression).	Data synthesis p8-9
	13f	Describe any sensitivity analyses conducted to assess robustness of the synthesized results.	n/a
Reporting bias	14	Describe any methods used to assess risk of bias due to missing results in a synthesis (arising from reporting biases).	Data synthesis

For peer review only - http://bmjopen.bmj.com/site/about/guidelines.xhtml

PRISMA 2020 Checklist

Section and Topic	Item #	Checklist item	Location where item is reported
assessment			p8-9
Certainty assessment	15	Describe any methods used to assess certainty (or confidence) in the body of evidence for an outcome.	n/a
**RESULTS**			
Study selection	16a	Describe the results of the search and selection process, from the number of records identified in the search to the number of studies included in the review, ideally using a flow diagram.	Results p9, fig1
	16b	Cite studies that might appear to meet the inclusion criteria, but which were excluded, and explain why they were excluded.	Included studies p9
Study characteristics	17	Cite each included study and present its characteristics.	Supp file2
Risk of bias in studies	18	Present assessments of risk of bias for each included study.	Supp file3
Results of individual studies	19	For all outcomes, present, for each study: (a) summary statistics for each group (where appropriate) and (b) an effect estimate and its precision (e.g. confidence/credible interval), ideally using structured tables or plots.	n/a
Results of syntheses	20a	For each synthesis, briefly summarise the characteristics and risk of bias among contributing studies.	Results/Themes p9-16
	20b	Present results of all statistical syntheses conducted. If meta-analysis was done, present for each the summary estimate and its precision (e.g. confidence/credible interval) and measures of statistical heterogeneity. If comparing groups, describe the direction of the effect.	n/a
	20c	Present results of all investigations of possible causes of heterogeneity among study results.	n/a
	20d	Present results of all sensitivity analyses conducted to assess the robustness of the synthesized results.	n/a
Reporting biases	21	Present assessments of risk of bias due to missing results (arising from reporting biases) for each synthesis assessed.	n/a
Certainty of evidence	22	Present assessments of certainty (or confidence) in the body of evidence for each outcome assessed.	n/a
**DISCUSSION**			
Discussion	23a	Provide a general interpretation of the results in the context of other evidence.	Discussion p16-17
	23b	Discuss any limitations of the evidence included in the review.	Discussion p16-17
	23c	Discuss any limitations of the review processes used.	Discussion p16-17
	23d	Discuss implications of the results for practice, policy, and future research.	Discussion p16-17
**OTH ER INFORMATION**			
Registration and protocol	24a	Provide registration information for the review, including register name and registration number, or state that the review was not registered.	Cover page p1
	24b	Indicate where the review protocol can be accessed, or state that a protocol was not prepared.	PROSPERO
	24c	Describe and explain any amendments to information provided at registration or in the protocol.	PROSPERO

For peer review only

PRISMA 2020 Checklist

Section and Topic	Item #	Checklist item	Location where item is reported
Support	25	Describe sources of financial or non-financial support for the review, and the role of the funders or sponsors in the review.	Cover page p1
Competing interests	26	Declare any competing interests of review authors.	p18
Availability of data, code and other materials	27	Report which of the following are publicly available and where they can be found: template data collection forms; data extracted from included studies; data used for all analyses; analytic code; any other materials used in the review.	Supplementary files

From: Page MJ, McKenzie JE, Bossuyt PM, Boutron I, Hoffmann TC, Mulrow CD, et al. The PRISMA 2020 statement: an updated guideline for reporting systematic reviews. BMJ 2021;372:n71. doi: 10.1136/bmj.n71

For more information, visit: http://www.prisma-statement.org/

For peer review only

BMJ Open

A qualitative systematic review of general practitioners' (GPs') views and experiences of providing postnatal care.

Journal:	*BMJ Open*
Manuscript ID	bmjopen-2022-070005.R1
Article Type:	Original research
Date Submitted by the Author:	07-Mar-2023
Complete List of Authors:	Macdonald, Clare; University of Birmingham, Institute of Applied Health Research<br>MacGregor, Becky; University of Warwick, Unit of Academic Primary Care<br>Hillman, Sarah ; University of Warwick, Unit of Academic Primary Care<br>MacArthur, Christine; University of Birmingham, Institute of Applied Health Research<br>Bick, Debra; University of Warwick<br>Taylor, Beck; University of Birmingham, Institute of Applied Health Research
**Primary Subject Heading**:	General practice / Family practice
Secondary Subject Heading:	Obstetrics and gynaecology, Qualitative research
Keywords:	REPRODUCTIVE MEDICINE, QUALITATIVE RESEARCH, PRIMARY CARE, Maternal medicine < OBSTETRICS, Reproductive medicine < GYNAECOLOGY, Primary Health Care

SCHOLARONE™
Manuscripts

I, the Submitting Author has the right to grant and does grant on behalf of all authors of the Work (as defined in the below author licence), an exclusive licence and/or a non-exclusive licence for contributions from authors who are: i) UK Crown employees; ii) where BMJ has agreed a CC-BY licence shall apply, and/or iii) in accordance with the terms applicable for US Federal Government officers or employees acting as part of their official duties; on a worldwide, perpetual, irrevocable, royalty-free basis to BMJ Publishing Group Ltd ("BMJ") its licensees and where the relevant Journal is co-owned by BMJ to the co-owners of the Journal, to publish the Work in this journal and any other BMJ products and to exploit all rights, as set out in our licence.

The Submitting Author accepts and understands that any supply made under these terms is made by BMJ to the Submitting Author unless you are acting as an employee on behalf of your employer or a postgraduate student of an affiliated institution which is paying any applicable article publishing charge ("APC") for Open Access articles. Where the Submitting Author wishes to make the Work available on an Open Access basis (and intends to pay the relevant APC), the terms of reuse of such Open Access shall be governed by a Creative Commons licence – details of these licences and which Creative Commons licence will apply to this Work are set out in our licence referred to above.

Other than as permitted in any relevant BMJ Author's Self Archiving Policies, I confirm this Work has not been accepted for publication elsewhere, is not being considered for publication elsewhere and does not duplicate material already published. I confirm all authors consent to publication of this Work and authorise the granting of this licence.

For peer review only

A qualitative systematic review of general practitioners' (GPs') views and experiences of providing postnatal care.

Authors

1. Clare Macdonald. Institute of Applied Health Research, University of Birmingham, Birmingham, B15 2TT. UK. CCM011@student.bham.ac.uk (corresponding author)
2. Becky MacGregor. Unit of Academic Primary Care, University of Warwick, Coventry, CV4 7AL. UK.
3. Sarah Hillman. Unit of Academic Primary Care, University of Warwick, Coventry, CV4 7AL. UK.
4. Christine MacArthur. Institute of Applied Health Research, University of Birmingham, Birmingham, UK. B15 2TT. https://orcid.org/0000-0003-0434-2158
5. Debra Bick. University of Warwick, Coventry, West Midlands, CV4 7AL. UK. https://orcid.org/0000-0002-8557-7276
6. Beck Taylor. Institute of Applied Health Research, University of Birmingham, Birmingham, UK. B15 2TT. https://orcid.org/0000-0002-3559-7922

Word count (excluding title page, abstract, references, figures and tables): 5210

Abbreviations: CM - Clare Macdonald, BM - Becky MacGregor, SH - Sarah Hillman, BT - Beck Taylor

PROSPERO registration: CRD42021268982

Where we use the terms 'woman' or 'women' in the context of their postnatal care, this is inclusive of all people who have given birth even if they do not identify as women.

For peer review only

ABSTRACT

Objectives

Develop an understanding of the views and experiences of GPs about their role in postnatal care, including barriers and facilitators to good care, and timing and content of planned postnatal checks.

Design

Qualitative systematic review.

Data sources

Electronic database searches of MEDLINE, EMBASE, CINAHL, PubMed, Web of Science, PsychINFO from January 1990 – September 2021. Grey literature and guideline references from National Institute of Health and Care Excellence, World Health Organization, International Federation of Gynecology and Obstetrics, Royal College of General Practitioners, Royal College of Obstetrics and Gynaecology.

Inclusion criteria

Papers reporting qualitative data on views and experiences of GPs about postnatal care, including discrete clinical conditions in the postnatal period. Papers were screened independently by two reviewers and disputes resolved by a third reviewer.

Quality appraisal

The CASP (Critical Appraisal Skills Programme) checklist was used to appraise studies.

Data extraction and synthesis

Thematic synthesis involving line-by-line coding, generation of descriptive then analytical themes was conducted by the review team. The COM-B model was used to develop analytical themes.

Results

20 reports from 18 studies met inclusion criteria. Studies were published from 2008-2021, reporting on 469 GPs. 13 were from UK or Australia. Some also reported views of non-GP participants. The clinical focus of studies varied, for example: perinatal mental health, postnatal contraception. Five themes were generated, four mapped to COM-B: psychological capability, physical opportunity, social opportunity and motivation. One theme was separate from the COM-B model: content and timing of postnatal checks. Strong influences were in physical and social opportunity, with time and organisation of services being heavily represented. These factors sometimes influenced findings in the motivation theme.

Conclusions

GPs perceived their role in postnatal care as a positive opportunity for relationship building and health promotion. Addressing organisational barriers could impact positively on GPs' motivation to provide the best care.

PROSPERO Registration Number: 268982

For peer review only - http://bmjopen.bmj.com/site/about/guidelines.xhtml

STRENGTHS AND LIMITATIONS

- This review synthesised the views and experiences of GPs about postnatal care, including its entire remit
- The search strategy was comprehensive, including grey literature and citation searches
- Only reports in English were included so some potentially relevant studies could have been overlooked
- Some included studies reported on non-GP participants in addition to GPs and it was not always possible to be certain which data was attributable to GPs

For peer review only

For peer review only - http://bmjopen.bmj.com/site/about/guidelines.xhtml

BACKGROUND

UK primary health care is generally organised and provided by practices who operate as independent contractors to the National Health Service (NHS), delivering 'General Medical Services'[1] to their registered patients. Since 2020, as part of this contract, providers in England are required to offer all women a postnatal check appointment 6-8 weeks after birth.[2] Community postnatal care, at home following birth, is multidisciplinary; as a minimum, midwives, health visitors and general practitioners (GPs) are typically involved. GPs see women for unplanned care in the postnatal period and for the routine 6–8-week check. Historically, in the UK, postnatal care provided after discharge from hospital has been an area of low priority in maternity services. Successive reports have found that women's experiences are often poor,[3,4] measurable outcomes such as breastfeeding rates are low[5] and even national guidance contextualises recommendations by describing postnatal care as a 'Cinderella subject'.[6] This is in spite of the postnatal period being a high risk time for maternal mortality and the unplanned maternal readmission rate being around 3.3%.[7]

A multidisciplinary, community-based postnatal care model is not unique to the UK. A systematic review of guidelines of community postnatal care found comparable recommendations from Australia and USA.[8] More recently, Canadian public health guidance included similar principles and themes and, similar to UK, stated that multiple healthcare professionals will be involved with postpartum care.[9] In its position statement from 2021, the Royal Australian College of General Practitioners stated that there were gaps in evidence for the role of GPs in maternity care with more research needed.[10]

UK GPs have become less involved in routine maternity care over time, markedly declining since the 1990s when intrapartum involvement dramatically reduced.[11] GPs are now often absent from antenatal care with women self-referring to NHS midwifery services, and midwives providing antenatal care even for women who subsequently require an obstetrician.[12] GPs have generally continued seeing women routinely around 6-8 weeks postnatally even when from 2004-2020 they had no contractual obligation to do so.[13] The quality was, however, variable; a 2017 report found women often felt rushed and 42% of those with emotional or mental health problems did not have this identified by their GP or other health professional.[14] This report called for designated funding for the GP postnatal check and the Royal College of General Practitioners (RCGP) echoed this.[15] In 2020, the review of the NHS England GP contract included specific funding for the GP 6-8 week.[2] Soon after this, the National Institute for Health and Care Excellence (NICE) published its updated Postnatal Care Guideline[6] specifying that the check should be done by a GP.

For peer review only - http://bmjopen.bmj.com/site/about/guidelines.xhtml

The volume and variety of topics that should be covered in the maternal postnatal check, according to NICE is vast, even with no patient-related complexity. Factors such as mode of birth, intrapartum complications and co-morbidities can affect the content of the consultation. Crucial to informing GPs' knowledge and understanding of the complexity of women's postnatal care requirements is information about their antenatal and intrapartum health and care provided by the hospital. NICE recommends that communication between secondary (i.e. hospital) and primary care is prompt and effective, including a plan for ongoing care and conditions that require long-term management. Current arrangements for the maternal postnatal check do not distinguish formally between women who require more complex, or additional follow-up due to health and/or potential social care complications from those who do not.

To the authors' knowledge, no previous review has evaluated the views and experiences of GPs on postnatal care, incorporating the various clinical topics that form the 6-8 week maternal check as defined by NICE. There are examples of qualitative systematic reviews on discrete postnatal care topics, for example gestational diabetes[16] and promotion of a healthy lifestyle in postpartum women,[17] but these do not provide an in-depth qualitative synthesis of the views and experiences of GPs encompassing the whole remit of postnatal care. The Cochrane Collaboration have planned a qualitative systematic review of factors influencing the provision of postnatal care by health workers,[18] but this will also include secondary and tertiary care settings with no specific focus on GPs' perspectives. Women's experiences are better documented than GPs' experiences and suggest inconsistencies in provision of care.[19] Given the broad scope and variability in quality of the GP 6-8 week maternal postnatal check, in order to understand the dynamic of these consultations and potential practitioner-based facilitators or barriers, it is necessary to understand the contextual and behavioural basis that potentially impacts the provision of postnatal care by GPs.

AIMS

This qualitative systematic review aimed to answer four research questions:

1. What do GPs say is, or should be, the content of the routine 6-8 week check (including non-clinical topics such as social complexity)
2. At what point in time do GPs say that they routinely review women after birth, and what timing do they think is optimal?
3. What do GPs see as the facilitators and barriers to providing high quality postnatal care in primary care settings?
4. What do GPs perceive to be their role in postnatal care, and what role do they say other members of the primary care team have, or should have?

For peer review only - http://bmjopen.bmj.com/site/about/guidelines.xhtml

METHODS

The review protocol was registered on the international prospective register of systematic reviews (PROSPERO) (Registration Number 268982).

Inclusion and exclusion criteria

The PerSPecTIF framework[20] was used to develop and define the detail of the research questions, and therefore eligibility criteria for inclusion of studies (Table 1). Given variability in provision of postnatal care in primary care, particularly the postnatal check, this framework was chosen with the aim of achieving a better understanding of context and acceptability from the perspectives of individuals about care provision. There was no intervention evaluated, or comparison made.

For peer review only

For peer review only - http://bmjopen.bmj.com/site/about/guidelines.xhtml

Table 1: PerSPecTIF Table of inclusion and exclusion criteria

PerSPecTIF	Inclusion	Comments
**Per**spective	General practitioners<br><br>General practitioner specialty trainees (GPSTs) (sometimes termed registrars)<br><br>Primary care physicians	Exclude hospital physicians / obstetricians / paediatricians<br><br>Exclude other healthcare workers e.g. midwives, health visitors, pharmacists
**S**etting	Primary care	Exclude secondary care settings
**P**henomenon of interest	Postnatal care of women and routine postnatal checks for women	Including the following defined clinical aspects of postnatal care<br>- Postnatal contraception<br>- Postnatal mental illness<br>- Gestational diabetes<br>- Hypertensive disorders of pregnancy<br>- Urinary incontinence<br>- Pelvic organ prolapse<br>Including, but not limited to other relevant topics such as postnatal infections (mastitis, perineal wound infection, caesarean section wound infection), breastfeeding related problems.<br><br>Including health promotion, social issues or concerns, social complexity, social prescribing
**E**nvironment	Community settings providing planned (routine review or follow-up) and unplanned postnatal care from any GPs	
**(C)**omparison	No comparison	
**T**iming	The postnatal period, encompassing the first 2 years after giving birth (because perinatal mental health services accept referrals up until 2 years after birth)	
**F**indings	In relation to the views and experiences of GPs, with relevance to GPs, policy makers, medical educators	

Information Sources

Database searches of MEDLINE, EMBASE, CINAHL, PubMed, Web of Science and PsychINFO were carried out in September 2021. Reference lists of included studies were reviewed for other potential studies meeting inclusion criteria, as were references from relevant grey literature including guidance from NICE, World Health Organization (WHO), International Federation of Gynecology and Obstetrics (FIGO), RCGP and Royal College of Obstetrics and Gynaecology (RCOG). Citation searching of included publications was completed. Grey literature and citation searches were conducted in March 2022.

Search Strategy

Searches were limited to publications where full text was available in English. Studies from before 1990 were excluded since UK GP involvement in maternity services became more confined to postnatal care from that time. Studies with populations from low and middle income countries[21] were excluded to maintain relevance to UK setting. The search strategy was piloted to ensure it captured key studies known to reviewers. (see supplementary file : Supp1)

Screening and data extraction

After removal of duplicates, titles and abstracts of all records were independently screened by two reviewers (CM and SH or BM). A third reviewer (BT) screened records where there was disagreement. A standardised data extraction proforma was developed to extract consistent, relevant information from studies including year of publication, setting, and analysis methodology. Data were extracted in line with the approach described by Thomas and Harden[22] whereby anything reported as 'findings', 'results' or equivalent were included in the analysis. Data from non-GP participants were not extracted. Data included themes, descriptions of themes and participant quotes. Full texts, including abstracts were checked for anything that could be considered a study finding. CM and SH or BM independently completed data extraction for a third of the included studies and there was a high degree of consistency, CM then completed data extraction for the remaining studies.

Data Synthesis

The thematic synthesis approach outlined by Thomas and Harden[22] was used as the basis for data synthesis. After multiple readings to gain familiarity, CM imported extracted data into NVivo12 software. Line by line coding was undertaken by CM, with BT independently coding a third of data and consistency of approach confirmed. CM and BT, in discussion with other review team members developed descriptive codes which was inductive, but with reference to the research questions. The data synthesis process was iterative and collaborative, taking place over several weeks with continual reference to the original studies and input from the review team to ensure that interpretation reflected the original studies. The review team remained open to the possibility of building the

For peer review only - http://bmjopen.bmj.com/site/about/guidelines.xhtml

analytical themes around pre-existing frameworks or models, depending on the nature of the data and the descriptive themes identified.

Patient and Public Involvement

The focus of this study was informed by feedback from the Applied Research Collaboration, West Midlands (ARC-WM) PPIE group that experiences of postnatal care, including the GP check were sometimes poor. This review was completed as part of a wider project around improving the GP postnatal check for which the ARC-WM PPIE group have regularly contributed.

RESULTS

Included studies

2693 unique references were identified in database searches and following screening, 23 full-text reports were assessed for eligibility by CM. The review team agreed the final set of included studies. 17 reports describing 15 studies met inclusion criteria from this process. One excluded report was an integrative review on physician perspectives of their role in perinatal mental health.[23] Studies included in this report were screened and two of these met eligibility criteria. Reasons for exclusion from the database searches were incorrect phenomenon of interest[24, 25] or incorrect perspective.[26-30] Three studies from references or citations of eligible reports met the inclusion criteria and were also included. In total, 20 reports were eligible, reporting on 18 studies (Figure 1).[31-50]

Studies were published between 2008 and 2021 representing views and experiences of 469 GPs. Nine were UK studies, four Australian, and the remainder from other countries, all with some degree of similarity to the UK postnatal care model. Twelve studies included other clinicians and GPs (e.g. health visitors, obstetricians), six included GPs only. As anticipated, studies focused on a range of postnatal care topics, or postnatal care overall. Most frequent were perinatal mental health (n=7) and follow-up of gestational diabetes (n=4). The remainder investigated postnatal care generally, follow-up of hypertension or pre-eclampsia, behaviour change opportunities, contraception and prescribing for breastfeeding women. Two studies reported qualitative analysis of survey results, others used various forms of interview for data collection; the inclusion of qualitative analysis of survey data explains the large number of participants overall. (see supplementary file: Supp2)

Quality appraisal

Quality appraisal was undertaken using the Critical Skills Appraisal Programme (CASP) Qualitative Checklist.[51] Studies were not excluded on methodological merit, but CASP was used to formally understand their integrity and reliability which aided interpretation of findings during the synthesis. CM completed the CASP assessment, with oversight from the review team. Most studies fulfilled all

For peer review only - http://bmjopen.bmj.com/site/about/guidelines.xhtml

or nearly all the checklist criteria, the area with least compliance being consideration of the relationship between researcher and participants which was only fulfilled in five studies. (see supplementary file: Supp3)

Themes

16 descriptive themes were identified (see supplementary file: Supp4). The final stage of thematic synthesis involved identifying analytical themes; moving beyond a descriptive summary of aggregated findings. One descriptive theme related specifically to two of the research questions – content and timing of the postnatal check. This did not generate new, analytical findings and is summarised descriptively. The remainder of the descriptive themes related to influences, barriers and facilitators to GPs in providing postnatal care. When reviewing and discussing these descriptive themes, to move to analytical themes, the research team reflected that these factors could in fact be mapped to the COM-B (Capability, Opportunity, Motivation – Behaviour) model; [52] this became the basis upon which the final analytical stage was completed. The descriptive themes were reviewed with reference to COM-B and mapped to relevant aspect of the model. The final themes were therefore: content and timing of postnatal checks (not mapped to COM-B), psychological capability, physical opportunity, social opportunity and motivation (no descriptive themes were mapped to the COM-B domain of 'physical capability'). (Figure 2). (see supplementary file: Supp5)

Content and timing of postnatal checks

Most studies mentioned the content of a routine postnatal check, or the typical remit of GPs' interactions with women.[31, 32, 35-37, 39-42, 44-46, 48-50] A broad range of specific clinical topics were cited as falling under GP responsibility, including asking about complications, such as hypertension or diabetes, more universal issues such as mental health, bleeding, pain, wound healing, fever, breastfeeding, sleep, resumption of sexual intercourse, contraception, future pregnancy plans, advice about exercise, diet and weight loss, and reminders about cervical screening. There was a strong sense that GPs perceived the routine postnatal check as holistic, with references to building a relationship with the woman's wider family[32, 44, 49] and preventative medicine:[37, 45, 46, 49]

> *"The GPs interviewed did not see their role limited to routine checks and managing illness. Instead they thought their responsibility extended to assessing how the family was functioning and providing anticipatory guidance and education when appropriate."*[32]

GPs' views on when a routine postnatal check should happen, and whether there should be more than one planned contact, were not commonly discussed. Two studies made specific reference to this[37, 44] without consensus regarding the optimal time; the earliest time discussed was a review within two weeks after birth[32] and no studies described a recommendation later than 8 weeks. There was

For peer review only - http://bmjopen.bmj.com/site/about/guidelines.xhtml

also a suggestion that the timing was woman-dependent and that 6 weeks may be right for some, but not all women. Similarly, the duration of the appointment was variable depending on the needs and complexity of the woman.

> *"six of the GPs felt that it [6 weeks] was potentially too late for young mothers: "I guess you could argue it's potentially a bit late for some women. I can certainly think of one or two quite young women who have got pregnant very quickly after having the first baby, and you wonder whether you might in some cases miss it." [GP11]"*[42]

Influences on GPs' approach to postnatal care:

1. Psychological capability

Psychological capability encompasses an individual's mental functioning, including understanding and memory.[52] A broad range of knowledge was found to be required to provide postnatal care which could be explained by the variety of clinical topics included, but one study focusing on postpartum care in general found good self-reported knowledge of a basic postpartum review:

> *"Most PCPs* [primary care physicians] *claimed to know the basic clinical examination of a postpartum woman. They were aware on the assessment of lochia cessation, wound recovery, pain control, screening for post-natal depression, breastfeeding, and preventive care, such as cervical cancer screening and contraception advice."*[46]

This was accompanied by acknowledgement that having a good basic knowledge as a generalist meant it was sometimes difficult to be expert in discrete areas:

> *"Participants acknowledged that the difficulty for GPs is that they offer a generalist service making it difficult for them to have expertise in all areas including PMH* [perinatal mental health]*: 'You are kind of jack-of-all-trades, you are master of none'"*[44]

There was overlap between psychological capability and physical opportunity in that GPs often referred to their use or awareness of resources such as guidelines when addressing the issue of their knowledge. Recall and interpretation of guidelines was sometimes poor.

> *"…you're not really sure what the responsibility is or what the frequency with which you should be checking it* [blood pressure] *is, and I don't think that the NICE guidance is so clear on that. (Participant 11—General Practitioner)"*[31]

Some knowledge gaps were identified in studies which explored specific topics, for example awareness of the condition itself[47], how a condition impacts the woman's mental health[48] and

For peer review only - http://bmjopen.bmj.com/site/about/guidelines.xhtml

For peer review only

future risks.[50] These findings across different conditions demonstrated the vast spectrum of pathology that GPs may come across postnatally and highlights that knowledge of conditions was not universal.

> *"Some HCPs* [healthcare professionals] *were not aware of PNA* [perinatal anxiety] *as a specific diagnosis and were uncertain that PNA existed as a distinct clinical entity: 'I mean it's a concept that I'm really not that much aware of either professionally or from reading.'"*[47]

The knowledge required to fulfil the broad remit of postnatal care was extensive and GPs expressed they had a good basic grasp of core topics but sometimes lacked knowledge of specific clinical areas, including difficulty understanding, accessing, or interpreting guidelines.

2. Physical opportunity

This theme describes environmental and physical resources and context with reference to the action or behaviour in question[52] and was represented by all studies.[31-50] Issues such as length of time available, guidelines and screening tools, and information supplied were frequently discussed. Some elements that were representative of both physical and social opportunity are summarised here.

GPs relied on information received from other services (usually hospital) about antenatal and intrapartum journeys. Information was sometimes inadequate, whether about a specific complication or care in general. There was a desire to have improved information, with frustration expressed by words such as 'disappointing', and descriptions of difficulty accessing relevant information quickly.

> *"GPs also identified the need for clarity and explicit recommendations for postnatal follow-up, indicating that the process and communication could be improved: "I would be keen to do a little bit more if it was simple and clear." [GP9]"*[41]

Guidelines, toolkits and screening tools were cited as resources known to or used by GPs as part of their clinical practice or continuing professional development. There was criticism of the clarity, accessibility and usability of guidelines across the different clinical topics which were out of the control of individual GPs.

> *"Non-availability of easily accessible, evidence based, up to date information on medicines in breastfeeding was mentioned. GPs often mentioned that their sources of information were conflicting and often "over cautious"."*[38]

Exposure to perinatal learning experiences and training were described as variable, in terms of how much training GPs had overall, and that some clinical topics such as breastfeeding were not as well

For peer review only - http://bmjopen.bmj.com/site/about/guidelines.xhtml

covered as others.

> *"Participants recalled limited information related to PMH* [perinatal mental health] *as the focus of their specialist training was on obstetric and medical topics and in terms of practical experience: 'It can be luck of the draw what you do get exposed to' (P1)."*[44]

There was no clear or consistent view on how long was allocated or needed for a postnatal check. The complexity of the woman impacted on how long the appointment would take, and therefore whether all aspects of the check were afforded adequate time. Moreover, there was sometimes a tendency to avoid certain clinical topics or tasks due to a lack of time, or fear of something time-consuming emerging. GPs would sometimes arrange a follow-up appointment if not everything could initially be covered.

> *"Most felt that a more realistic time per consult should last from 15 to 20 min, compared to the current 5 to 10 min in real-time clinical practice. The limited time for consult often reduced the comprehensiveness and scope of postpartum issues covered within the postpartum visit. Consequently, the PCPs* [primary care physicians] *tended to avoid time-consuming tasks such as mental health assessment or discussing contraception options with the mothers."*[46]

In UK studies in particular, there was frustration with organisational changes meaning that health visitors and GPs were no longer co-located, impacting negatively on communication. There were positive descriptions of examples of good care when services were organised as well run multi-disciplinary teams. GPs sometimes felt disconnected by their reduced role in antenatal care which impacted on their ability to provide continuity. Organisational changes around GP and health visitor working were described as reducing opportunities for long-term relationships. However, GPs reflected positively on their professional autonomy to offer follow-up appointments when indicated. There was a recognition of the difficulties that women could face in accessing primary care services.

> *"practitioners reported that ongoing NHS reform had resulted in health visitors being moved out of general practice and into centralized services (often in non-NHS settings), a development universally regarded as deleterious to multiagency team work and delivery of effective perinatal mental healthcare"*[36]

Overall, many aspects of physical opportunity were consistently cited as important to GPs. The wide range of elements identified reflected the complexities of postnatal care itself and the intricacies of service organisation. That GPs sometimes had little or no influence over these factors impacted on

For peer review only - http://bmjopen.bmj.com/site/about/guidelines.xhtml

their approach to patients with a sense that they wanted more time to be made available for the women with more complex issues, and better resources to complement their clinical experience.

3. Social Opportunity

Six descriptive themes were relevant to social opportunity, which encompasses aspects of social norms and culture involving interactions between different people.[52] There was sometimes uncertainty from GPs about who was responsible for setting the agenda in the postnatal check or ensuring appropriate follow-up. Some GPs described a flexible approach, covering both the woman's and the GP's agenda.

> *""You've got to be quite fluid....What is it that they've come to see you about? I've got to meet that need primarily. I've got to discharge my need which is keeping them safe and running through all the possible scenarios of something going terribly wrong… anything extra that I can plug in terms of education, that's a bonus." (RGP7)"*[32]

Also influencing the nature of the consultation, was GPs citing the baby as a distraction, impacting on their care of the woman.

> *"The GPs reported that after delivery, the primary focus of care shifts from the woman with GDM to the baby."*[50]

GPs expressed a desire to understand the cultural and social background of women and how this might impact care they needed. Additionally, GPs sometimes contextualised potential barriers to compliance with care or recommendations within their perception of social norms about women finding it difficult to engage with their health and future risk.

> *"The attitudes of a mother to self-care and health were seen as a barrier. Denial of risk was perceived by some GPs to further hinder follow-up care."*[45]

Overlapping with organisational factors captured in the physical opportunity theme were GPs' perceptions of their own role, and roles of colleagues such as health visitors and midwives. GPs' minimal or absent involvement with women antenatally was cited as a reason for difficulties with postnatal care, or justification for some aspects of postnatal care being handled by someone else.

> *"A few GPs also referred in qualitative comments to their increasing distance from antenatal care due to organisational changes placing some midwives and health visitors outside practice surgeries: "I feel my role has been marginalised since joint working with health visitors has effectively stopped." (GP, survey respondent)"*[39]

For peer review only - http://bmjopen.bmj.com/site/about/guidelines.xhtml

This theme captured tensions GPs experienced when trying to balance conflicting consultation agendas, distractions, and evolving professional roles with their own beliefs about what women want or need postnatally.

4. Motivation

The COM-B Model distinguishes between reflective and automatic motivation[52], but in the analysis, motivational features were difficult to categorise as solely one of these (COM-B acknowledges that, for example, automatic processes can arise from reflection[53]) and therefore Motivation was combined as a single theme. The descriptive themes that mapped to this were 'Doctor-related factors' and 'Clinical decision making'. This theme captured GPs' perceptions about how they reached decisions, and insights into past professional or personal experiences that impacted their actions. Studies with a focus on mental health captured examples of reticence to arrive at a diagnosis, a desire to normalise aspects of perinatal mental illness, including dismissing symptoms as being part of normal life.[33-35, 39, 43]

> "[with reference to a case vignette of a traumatic birth] *"Without taking anything away from the trauma and distress suffered, there is a chance that this also could be something this woman could work through and doesn't necessarily see a doctor about and manages to recover from quite well without needing medical intervention." GP 6 (Male)"*[43]

GPs sometimes resisted their instinct or known expectation to explore perinatal mental health issues for reasons such as desire to avoid awkwardness.

> *"'Can I be honest with you sometimes I wonder if you really want to open this can of worms and it's so much easier just to jolly along and check the BP, check the urine, check this and that and have them out the door and see the next patient' (P5)."*[44]

In some cases, social opportunity seemed to impact on motivation: the nature of relationships between healthcare professions or between GPs and women influenced the motivation of the GP to act in a certain way. For example, when continuity of care was not achieved, GPs were less motivated to be thorough:

> *"Some health professionals described consciously inhibiting disclosure in order not to be placed in this position citing lack of continuity of care as the reason: "Easier not to ask, if I'm not going to see her again." (L GP1)"*[34]

Some factors appeared to increase GPs' confidence in making a diagnosis or enabling disclosure of

For peer review only - http://bmjopen.bmj.com/site/about/guidelines.xhtml

symptoms such as involvement of others in decision making, use of clinical instinct, and patient insight into their symptoms.

> *"'Your antenna would be raised by people coming clean with you that there is something going on…a history… the usual kind of joy isn't there, they're, quiet' (P5)."*[44]

GPs' views about their roles most commonly fell within the social opportunity theme, but there were motivational influences whereby beliefs about their role could impact the nature of their interaction with the woman.

> *"Many considered their main purpose as treating physical health problems and not to promote health."*[49]

Overall, there was a reliance on experience and clinical acumen and a desire to share decision making with the woman and professional colleagues. This was combined with some resistance to completing a diagnostic process when pathology had been identified or even avoiding the diagnostic process altogether.

DISCUSSION

This review suggested wide ranging influences in GPs' overall approach to postnatal care, the strongest being physical and social opportunity, and that these issues impact on motivational factors. With regard to the timing of routine checks, it was perhaps surprising that little data made reference to this, given its lack of evidence base and therefore potential for uncertainty. There was, however, some uncertainty about whether 6-8 weeks was the ideal time, for example that it might be too late for contraceptive needs, but also an unquestioning acceptance that 6-8 weeks was the norm. This could be explained by 6-8 weeks being culturally embedded, being recommended in guidelines and coinciding with the planned infant physical examination and first immunisations. The absence of research to determine the optimal time for a routine postnatal check could explain why GPs lacked a clear sense of what might be ideal, and usual practice continuing without formal evaluation as to whether 6–8 weeks is appropriate to conclude a woman's maternity care. The content of postnatal care consultations, in terms of the clinical topics covered was described as wide reaching and was often influenced by GPs' professional experience or the woman's own agenda rather than formal clinical guidance. This introduced further variability because, for example, GPs had different levels of experience and weren't always able to influence their own clinical jobs or rotations, and women's confidence, knowledge, or ability to present their own agenda would vary significantly.

For peer review only - http://bmjopen.bmj.com/site/about/guidelines.xhtml

For peer review only

GPs perceived themselves as having a key role in the clinical care of women – and their families – in the postnatal period. In describing their perception of the roles of other healthcare professionals, they clearly expressed the benefits of multidisciplinary working, and the importance of the roles of others, particularly health visitor colleagues. It appeared that when there was physical proximity, or straightforward access to health visitors, GPs felt more able to provide better care. However, there was also reference to the difficulties arising from lost continuity with antenatal care falling outside of the GPs' remit, and complex women being managed in secondary care without clear communication. A key facilitator was the professional satisfaction and enjoyment GPs derived when seeing women postnatally, as part of their care for the whole family. However, they could be reluctant to provide a comprehensive postnatal check when constrained by time or perceived a risk of uncovering complexity by raising certain topics such as perinatal mental health. GPs viewed the task of providing care for women postnatally as more challenging when they lacked time, clear clinical guidance, and access to suitable communication from secondary care.

Limitations of this review included the heterogeneity between studies which was anticipated but resulted in the synthesis of data from studies about different discrete clinical topics. This was considered throughout the analysis, and findings from studies concerning postnatal care as a broad topic contained similar findings, giving some assurance to this approach. The inclusion of non-GP participants in some studies meant that during data extraction, it was sometimes difficult to be certain which data was from GPs. Although there are examples globally of similar postnatal care models to the UK, there are important differences that could influence the analysis, for example, studies from Australia and Singapore reference different needs of private and public patients which is not so relevant to the UK. Finally, whilst the included studies were all appraised against CASP criteria, one was a report not formally peer-reviewed.[39]

Utilisation of the COM-B model as part of the analysis was made after the team reflected that this model was able to neatly frame what the data were showing. Application of the COM-B model to the synthesis allowed for translation of findings to the model's associated Behaviour Change Wheel (BCW)[52] and therefore the generation of potential intervention or policy areas that apply to each behaviour. The strongest influences of social and physical opportunity revealed intervention categories of education, persuasion, incentivisation and coercion. GP practices have already been financially incentivised to offer women a postnatal check since the 2020 change to the NHS England GP contract, but this has not been accompanied by complementary interventions such as education or persuasion. It may be possible in the future to determine whether this intervention has improved postnatal check provision, but it will not measure quality. By targeting interventions recommended in

For peer review only - http://bmjopen.bmj.com/site/about/guidelines.xhtml

the BCW from the motivation and social opportunity themes (such as enablement, training, education), quality and consistency could be improved.

The postnatal period is one of marked health inequality.[7] This is a critical issue that requires further research to better understand causes, and intervention to overcome it. This review found minimal reference to differential risk between populations, except for one study which looked specifically at Black and ethnic minority women,[36] and higher risk groups were not well defined or explained in included studies. The suggestion within the physical opportunity theme that different women needed different lengths of time for a postnatal review may be important in addressing this inequality; if women at higher risk of morbidity or mortality could be identified, they could be given a longer appointment, or additional follow-up. From what is already know about disparities in outcomes for women with different social and medical backgrounds, it may be reasonable to extrapolate that adjusting routine care to meet needs has the potential to improve outcomes and reduce inequality.

Future research should address the issue of timing of the routine maternal postnatal check, both when it should happen, and how long it should take. These research areas should be approached with specific reference to groups known to be most at risk. In the UK context, the introduction of a mandatory maternal postnatal check to the GP contract should be evaluated, from the perspectives of women, but also GPs who provide it, so that its provision and quality are optimised.

For peer review only

For peer review only - http://bmjopen.bmj.com/site/about/guidelines.xhtml

Figure Legends

Figure 1: Study selection process

Figure 2: Themes mapped to COM-B

Contributorship statement

Clare Macdonald developed the protocol, carried out the searches, participated in the selection process and data extraction, undertook the coding process, led the analysis and drafted the manuscript. Becky MacGregor participated in the selection process and data extraction, contributed to the analysis, and contributed to and approved the manuscript. Sarah Hillman reviewed the protocol, participated in the selection process and data extraction, contributed to the analysis, and contributed to and approved the manuscript. Christine MacArthur reviewed and contributed to the protocol, contributed to the analysis, and contributed to and approved the manuscript. Debra Bick reviewed and contributed to the protocol, contributed to the analysis, and contributed to and approved the manuscript. Beck Taylor reviewed and contributed to the protocol, participated in the selection process, undertook the coding process, contributed to the analysis, and contributed to and approved the manuscript.

Competing interests

There are no competing interests for any author.

Funding

Clare Macdonald is funded by the National Institute for Health Research (NIHR) West Midlands Applied Research Collaboration. The views expressed are those of the author and not necessarily those of the NIHR or the UK Department of Health and Social Care.

Data sharing statement

There are no plans to store further data in an open repository but much of the review data has been submitted in supplementary files and the corresponding author can be contacted if there are any future requests for further data.

Ethics approval statement

This is a qualitative systematic review and ethics approval was not required. The ethical considerations and permissions in the studies included in the review were considered as part of the quality appraisal process.

For peer review only - http://bmjopen.bmj.com/site/about/guidelines.xhtml

REFERENCES

1. The National Health Service (General Medical Services Contracts) Regulations 2015 [Available from: https://www.legislation.gov.uk/uksi/2015/1862/regulation/3. Accessed 10/2022
2. NHS England BMA. Investment and Evolution - Update to the GP Contract Agreement 2021-23/24 2020 [Available from: https://www.england.nhs.uk/publication/investment-and-evolution-update-to-the-gp-contract-agreement-20-21-23-24/. Accessed 10/2022
3. The National Federation of Women's Institutes N. Support overdue: Women's experiences of maternity services. 2017 [Available from: https://www.thewi.org.uk/__data/assets/pdf_file/0009/187965/NCT-nct-WI-report-72dpi.pdf. Accessed 10/2022
4. Cumberlege J. Better Births. Improving outcomes of maternity services in England. A Five Year Forward. View for maternity care. 2016 [Available from: https://www.england.nhs.uk/wp-content/uploads/2016/02/national-maternity-review-report.pdf. Accessed 10/2022
5. Public Health England. Breastfeeding prevalence at 6 to 8 weeks after birth: Annual data statistical commentary 2019 to 2020 2021 [Available from: https://assets.publishing.service.gov.uk/government/uploads/system/uploads/attachment_data/file/957253/OFF_SEN_Annual_Breastfeeding_Statistical_Commentary_2019_2020.pdf. Accessed 10/2022
6. NICE. National Institute for Health and Care Excellence: Postnatal Care guideline (NG194) 2021 [Available from: https://www.nice.org.uk/guidance/ng194. Accessed 10/2022
7. MBRRACE-UK. Saving Lives, Improving Mothers' Care. Lessons learned to inform maternity care from the UK and Ireland Confidential Enquiries into Maternal Deaths and Morbidity 2016-18 2020 [Available from: https://www.npeu.ox.ac.uk/assets/downloads/mbrrace-uk/reports/maternal-report-2020/MBRRACE-UK_Maternal_Report_Dec_2020_v10_ONLINE_VERSION_1404.pdf. Accessed 10/2022
8. Haran C, van Driel M, Mitchell BL, et al. Clinical guidelines for postpartum women and infants in primary care-a systematic review. *BMC Pregnancy Childbirth.* 2014;14:51.
9. Public Health Agency of Canada. Family-centred maternity and newborn care: National guidelines 2021 [Available from: https://www.canada.ca/en/public-health/services/maternity-newborn-care-guidelines.html. Accessed 10/2022
10. Royal Austrailian College of General Practitioners. Maternity Care in General Practice. Position. 2021 [21.6.21]. Available from: https://www.racgp.org.au/advocacy/position-statements/view-all-position-statements/clinical-and-practice-management/maternity-care-in-general-practice#ref-num-10. Accessed 10/2022
11. Smith A. Shakespeare J. Dixon A. The role of GPs in maternity care – what does the future hold? : The King's Fund; 2010.
12. NICE. National Institute for Health and Care Excellence: Antenatal care guideline (NG201) 2021 [Available from: https://www.nice.org.uk/guidance/ng201. Accessed 10/2022
13. The National Health Service (General Medical Services Contracts) Regulations 2004 No. 291 [Available from: https://www.legislation.gov.uk/uksi/2004/291/contents/made. Accessed 10/2022
14. NCT. The Hidden Half: Bringing postnatal mental illness out of hiding 2017 [Available from: https://www.nct.org.uk/sites/default/files/related_documents/739-NCT-theHiddenHalf-report-150dpi_0.pdf. Accessed 10/2022
15. RCGP. GPs doing very best for new mothers but need more time and resources, says RCGP 2017 [Available from: https://www.rcgp.org.uk/about-us/news/2017/june/gps-doing-very-best-for-new-mothers-but-need-more-time-and-resources.aspx. Accessed 10/2022
16. Lithgow GE, Rossi J, Griffin SJ, et al. Barriers to postpartum diabetes screening: a qualitative synthesis of clinicians' views. *British Journal of General Practice*. 2021;71(707):E473-E82.

For peer review only - http://bmjopen.bmj.com/site/about/guidelines.xhtml

17. Makama M, Awoke MA, Skouteris H, et al. Barriers and facilitators to a healthy lifestyle in postpartum women: A systematic review of qualitative and quantitative studies in postpartum women and healthcare providers. *Obesity Reviews*. 2021;22(4):e13167.
18. Munabi-Babigumira S, Lewin S, Glenton C, et al. Factors that influence the provision of postnatal care by health workers: a qualitative evidence synthesis. *Cochrane Database of Systematic Reviews*. 2021(7).
19. NCT. NCT finds a quarter of new mothers are not asked about their mental health 2021 [Available from: https://www.nct.org.uk/about-us/media/news/nct-finds-quarter-new-mothers-are-not-asked-about-their-mental-health. Accessed 10/2022
20. Booth A, Noyes J, Flemming K et al. Formulating questions to explore complex interventions within qualitative evidence synthesis. *BMJ Global Health*. 2019;4(Suppl 1):e001107.
21. OECD. DAC List of ODA Recipients 2021 [Available from: https://www.oecd.org/dac/financing-sustainable-development/development-finance-standards/daclist.htm. Accessed 10/2022
22. Thomas J, Harden A. Methods for the thematic synthesis of qualitative research in systematic reviews. *BMC Medical Research Methodology*. 2008;8(1):45.
23. Noonan M, Doody O, Jomeen J,et al. Family physicians perceived role in perinatal mental health: an integrative review. *BMC Family Practice*. 2018;19(1):154.
24. Ibanez G, Zabar J, Cadwallader JS, et al. Views of general practitioners on indoor environmental health risks in the perinatal period. *Frontiers in Medicine*. 2015;2:32.
25. Weingarten MA, Reinitz A, Hart J. Attitudes to primary-care gynaecology among family physicians and gynaecologists in Israel. *Scandinavian Journal of Primary Health Care*. 1992;10(1):36-41.
26. Naughton F, Hopewell S, Sinclair L, et al. Barriers and facilitators to smoking cessation in pregnancy and in the post-partum period: The health care professionals' perspective. *British journal of health psychology*. 2018;23(3):741-57.
27. Kline CR, Martin DP, Deyo RA. Health consequences of pregnancy and childbirth as perceived by women and clinicians. *Obstetrics & Gynecology*. 1998;92(5):842-8.
28. Green A, Callaway L, McIntyre HD, et al. Diagnosing and providing initial management for patients with Gestational Diabetes: What is the General Practitioner's experience? *Diabetes Research & Clinical Practice*. 2020;166:108290.
29. Rivas K, Schmuhl NB, Taboada M, et al. Are Wisconsin resident physicians trained in immediate postpartum long acting reversible contraception? *Contraception.* 2021;104(4):372-6.
30. Kean LJ, Hamilton J, Shah P. Antidepressants for mothers: what are we prescribing? *Scottish Medical Journal*. 2011;56(2):94-7.
31. Bick D, Silverio SA, Bye A, et al. Postnatal care following hypertensive disorders of pregnancy: a qualitative study of views and experiences of primary and secondary care clinicians. *BMJ Open*. 2020;10(1):e034382.
32. Brodribb W, Zadoroznyj M, Dane A. The views of mothers and GPs about postpartum care in Australian general practice. *BMC Family Practice*. 2013;14:139.
33. Chew-Graham C, Chamberlain E, Turner K, et al. GPs' and health visitors' views on the diagnosis and management of postnatal depression: a qualitative study. *British Journal of General Practice*. 2008;58(548):169-76.
34. Chew-Graham CA, Sharp D, Chamberlain E, et al. Disclosure of symptoms of postnatal depression, the perspectives of health professionals and women: a qualitative study. *BMC Family Practice*. 2009;10(1):7.
35. Dijkhuis T, Bloem F, Kusters L, et al. Investigating the current knowledge and needs concerning a follow-up for long-term cardiovascular risks in Dutch women with a preeclampsia history: a qualitative study. *BMC Pregnancy and Childbirth*. 2020;20.
36. Edge D. Falling through the net - black and minority ethnic women and perinatal mental healthcare: health professionals' views. *Gen Hosp Psychiatry*. 2010;32(1):17-25.

For peer review only - http://bmjopen.bmj.com/site/about/guidelines.xhtml

37. Hewage SS, Singh SR, Chi C, et al. Health Care Providers' Perceptions of Responsibilities and Resources to Reduce Type 2 Diabetes Risk After Gestational Diabetes Mellitus. *Clin Diabetes.* 2018;36(2):160-7.
38. Jayawickrama HS, Amir LH, Pirotta MV. GPs' decision-making when prescribing medicines for breastfeeding women: Content analysis of a survey. *BMC Research Notes.* 2010;3:82.
39. Khan L. Falling through the gaps: perinatal mental health and general practice. www.centreformentalhealth.org.uk; 2015.
40. Kilgour C, Bogossian FE, Callaway L, et al. Postnatal gestational diabetes mellitus follow-up: Perspectives of Australian hospital clinicians and general practitioners. *Women & Birth: Journal of the Australian College of Midwives*. 2019;32(1):e24-e33.
41. Kilgour C, Bogossian F, Callaway L, et al. Experiences of women, hospital clinicians and general practitioners with gestational diabetes mellitus postnatal follow-up: A mixed methods approach. *Diabetes Research & Clinical Practice.* 2019;148:32-42.
42. Lunniss H, Cameron S, Chen ZE. Views of general practitioners on providing contraceptive advice and long-acting reversible contraception at the 6-week postnatal visit: a qualitative study. *Journal of Family Planning and Reproductive Health Care*. 2016;42(2):99-105.
43. Mortimer H, Habash-Bailey H, Cooper M, et al. An exploratory qualitative study exploring GPs' and psychiatrists' perceptions of post-traumatic stress disorder in postnatal women using a fictional case vignette. *Stress and Health*. 2021;38(3):544-555.
44. Noonan M, Doody O, O'Regan A, et al Irish general practitioners' view of perinatal mental health in general practice: a qualitative study. *BMC Family Practice*. 2018;19(1):196.
45. Pennington AVR, O'Reilly SL, Doris Y, et al. Improving follow-up care for women with a history of gestational diabetes: perspectives of GPs and patients. *Australian Journal of Primary Health*. 2017;23(1):66-74.
46. Poon Z, Lee ECW, Ang L, et al. Experiences of primary care physicians managing postpartum care: a qualitative research study. *BMC Family Practice*. 2021;22(1).
47. Silverwood V, Nash A, Chew-Graham CA, et al. Healthcare professionals' perspectives on identifying and managing perinatal anxiety: a qualitative study. *British Journal of General Practice.* 2019;69(688):E768-E76.
48. Sriranjan AN, Abrams R, Wong G, et al. Towards an understanding of GPs' viewpoint on diagnosing postnatal depression in general practice: a small-scale realist evaluation. *Primary Health Care Research and Development.* 2020;21.
49. Talbot H, Strong E, Peters S, et al. Behaviour change opportunities at mother and baby checks in primary care: a qualitative investigation of the experiences of GPs. *British Journal of General Practice*. 2018;68(669):e252-e9.
50. Timm A, Nielsen KK, Christensen U, et al. Healthcare Professionals' Perspectives on the Cross-Sectoral Treatment Pathway for Women with Gestational Diabetes during and after Pregnancy-A Qualitative Study. *Journal of Clinical Medicine*. 2021;10(4).
51. Critical Appraisal Skills Programme. CASP qualitative checklist 2019 [23.06.2021]. Available from: https://casp-uk.net/casp-tools-checklists/. Accessed 10/2022
52. Michie S, van Stralen MM, West R. The behaviour change wheel: A new method for characterising and designing behaviour change interventions. *Implementation Science*. 2011;6(1):42.
53. Michie S, West R. Behaviour change theory and evidence: a presentation to Government. *Health Psychology Review*. 2013;7(1):1-22.

For peer review only - http://bmjopen.bmj.com/site/about/guidelines.xhtml

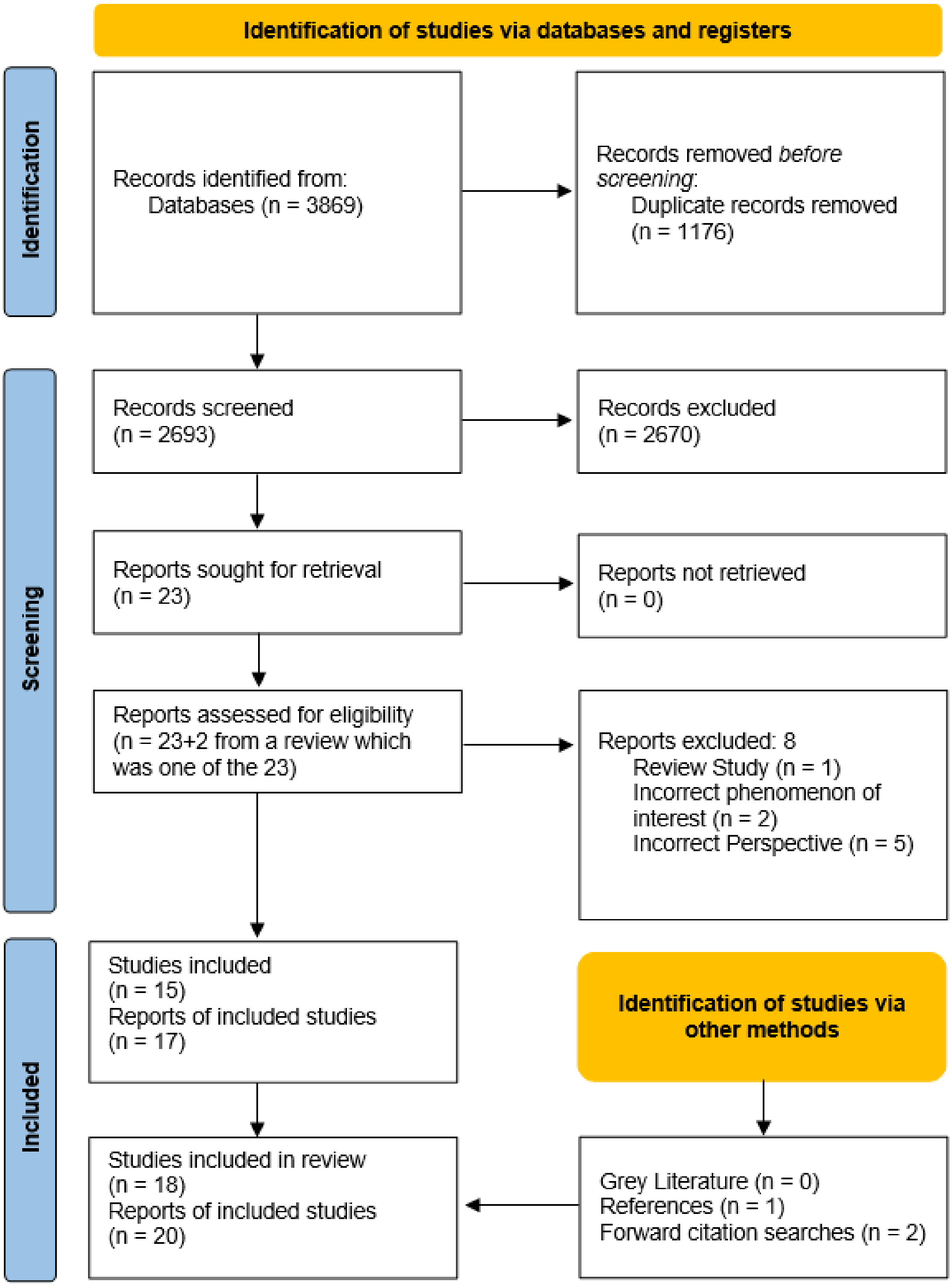
Identification of studies via databases and registers
Identification
Records identified from:
Databases (n = 3869)
Records removed *before screening*:
Duplicate records removed (n = 1176)
Screening
Records screened
(n = 2693)
Records excluded
(n = 2670)
Reports sought for retrieval
(n = 23)
Reports not retrieved
(n = 0)
Reports assessed for eligibility
(n = 23+2 from a review which was one of the 23)
Reports excluded: 8
Review Study (n = 1)
Incorrect phenomenon of interest (n = 2)
Incorrect Perspective (n = 5)
Included
Studies included
(n = 15)
Reports of included studies
(n = 17)
Identification of studies via other methods
Studies included in review
(n = 18)
Reports of included studies
(n = 20)
Grey Literature (n = 0)
References (n = 1)
Forward citation searches (n = 2)

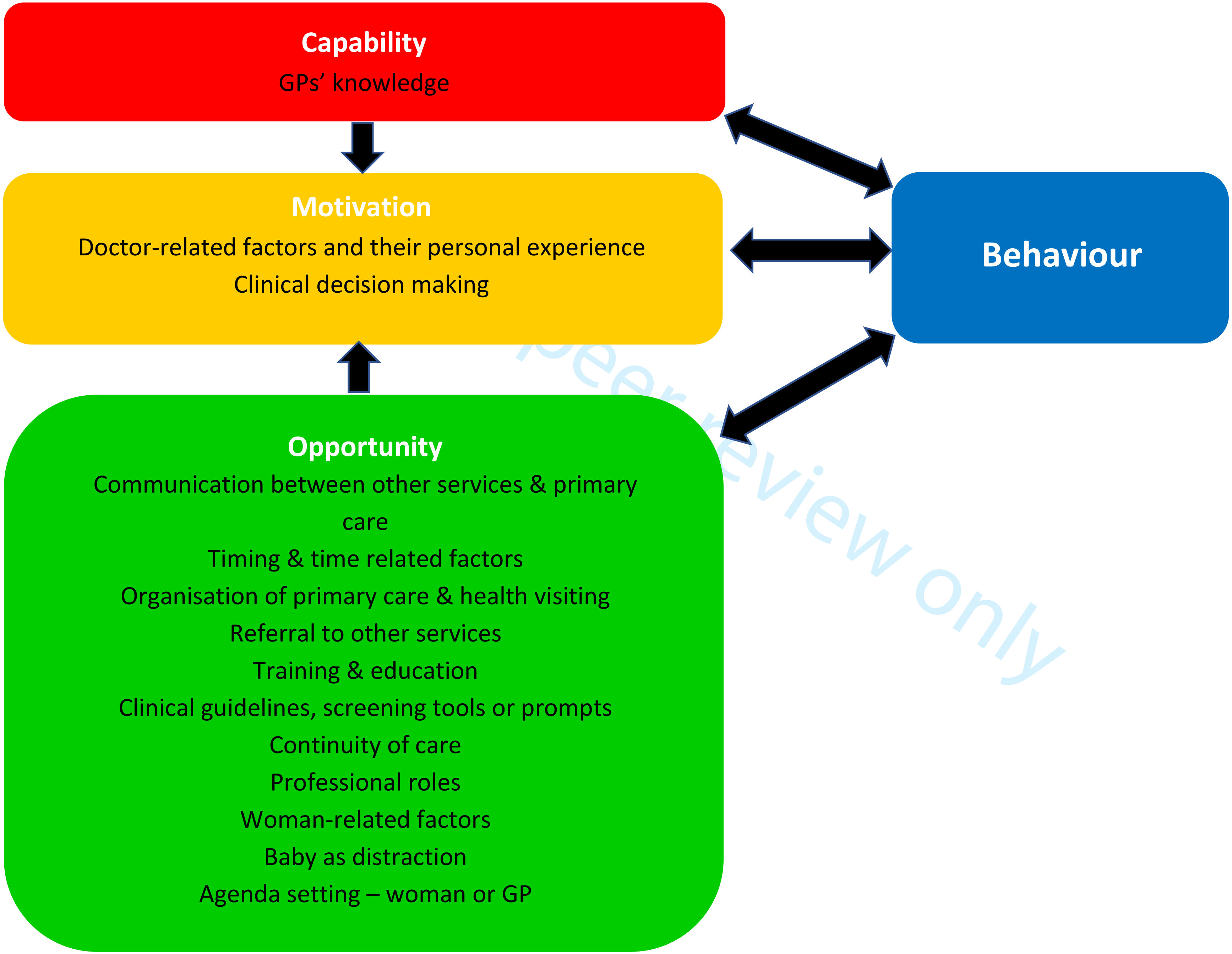

For peer review only - http://bmjopen.bmj.com/site/about/guidelines.xhtml

Supplementary File 1: Search Terms

**MEDLINE**	Ovid MEDLINE(R) and In-Process, In-Data-Review & Other Non-Indexed Citations <1946 to September 27, 2021><br><br>1 General Practitioners/ 8852<br>2 Primary Health Care/ 83832<br>3 Physicians, Primary Care/ 3919<br>4 Physicians, Family/ 16693<br>5 (general practitioner* or GP*).mp. [mp=title, abstract, original title, name of substance word, subject heading word, floating sub-heading word, keyword heading word, organism supplementary concept word, protocol supplementary concept word, rare disease supplementary concept word, unique identifier, synonyms] 252660<br>6 1 or 2 or 3 or 4 or 5 340751<br>7 Perinatal Care/ 5026<br>8 Postpartum Period/ 27536<br>9 Postnatal Care/ 6048<br>10 Maternal Health/ 1909<br>11 exp Puerperal Disorders/ 35531<br>12 Diabetes, Gestational/ 12317<br>13 Hypertension, Pregnancy-Induced/ 3775<br>14 exp Urinary Incontinence/ 34163<br>15 Pelvic Organ Prolapse/ 4269<br>16 (perinatal or postpartum or postnatal).mp. [mp=title, abstract, original title, name of substance word, subject heading word, floating sub-heading word, keyword heading word, organism supplementary concept word, protocol supplementary concept word, rare disease supplementary concept word, unique identifier, synonyms] 247345<br>17 7 or 8 or 9 or 10 or 11 or 12 or 13 or 14 or 15 or 16 312631<br>18 "Attitude of Health Personnel"/ or Attitude/ 176055<br>19 Perception/ 38727<br>20 (attitude* or belief* or opinions* or understanding* or viewpoint* or perspective* or perception* or view* or experience*).mp. [mp=title, abstract, original title, name of substance word, subject heading word, floating sub-heading word, keyword heading word, organism supplementary concept word, protocol supplementary concept word, rare disease supplementary concept word, unique identifier, synonyms] 3341799<br>21 18 or 19 or 20 3341799<br>22 6 and 17 and 21 649<br>Limited to 1990-2021 = 607
**PsychInfo**	APA PsycInfo <1967 to September Week 3 2021><br><br>1 General Practitioners/ 6045<br>2 Primary Health Care/ 19408<br>3 Physicians, Primary Care/ 0<br>4 Physicians, Family/ 0<br>5 (general practitioner* or GP*).mp. [mp=title, abstract, heading word, table of contents, key concepts, original title, tests & measures, mesh] 27828<br>6 1 or 2 or 3 or 4 or 5 44630<br>7 Perinatal Care/ 0<br>8 Postpartum Period/ 0

For peer review only

Supplementary File 1: Search Terms

	9 Postnatal Care/ 0<br>10 Maternal Health/ 0<br>11 exp Puerperal Disorders/ 0<br>12 Diabetes, Gestational/ 0<br>13 Hypertension, Pregnancy-Induced/ 0<br>14 exp Urinary Incontinence/ 2004<br>15 Pelvic Organ Prolapse/ 0<br>16 (perinatal or postpartum or postnatal).mp. [mp=title, abstract, heading word, table of contents, key concepts, original title, tests & measures, mesh] 42320<br>17 7 or 8 or 9 or 10 or 11 or 12 or 13 or 14 or 15 or 16 44299<br>18 "Attitude of Health Personnel"/ or Attitude/ 0<br>19 Perception/ 19909<br>20 (attitude* or belief* or opinions* or understanding* or viewpoint* or perspective* or perception* or view* or experience*).mp. [mp=title, abstract, heading word, table of contents, key concepts, original title, tests & measures, mesh] 1955569<br>21 18 or 19 or 20 1955569<br>22 6 and 17 and 21140<br>23 from 22 keep 1-3,5-137 136Limited to 1990 – 2021 = 136 (LIMITED TO 1990-2021)
**EMBASE**	Embase <1974 to 2021 September 27><br>1 General Practitioners/ 100397<br>2 Primary Health Care/ 71097<br>3 Physicians, Primary Care/ 106008<br>4 Physicians, Family/ 100399<br>5 (general practitioner* or GP*).mp. [mp=title, abstract, heading word, drug trade name, original title, device manufacturer, drug manufacturer, device trade name, keyword heading word, floating subheading word, candidate term word] 381855<br>6 1 or 2 or 3 or 4 or 5 444488<br>7 Perinatal Care/ 14860<br>8 Postpartum Period/ 35277<br>9 Postnatal Care/ 7813<br>10 Maternal Health/ 15058<br>11 exp Puerperal Disorders/ 45592<br>12 Diabetes, Gestational/ 19790<br>13 Hypertension, Pregnancy-Induced/ 11617<br>14 exp Urinary Incontinence/ 79211<br>15 Pelvic Organ Prolapse/ 15877<br>16 (perinatal or postpartum or postnatal).mp. [mp=title, abstract, heading word, drug trade name, original title, device manufacturer, drug manufacturer, device trade name, keyword heading word, floating subheading word, candidate term word] 336868<br>17 7 or 8 or 9 or 10 or 11 or 12 or 13 or 14 or 15 or 16 483411<br>18 "Attitude of Health Personnel"/ or Attitude/ 138747<br>19 Perception/ 135053<br>20 (attitude* or belief* or opinions* or understanding* or viewpoint* or perspective* or perception* or view* or experience*).mp. [mp=title, abstract, heading word, drug trade name, original title, device manufacturer, drug

For peer review only

Supplementary File 1: Search Terms

	manufacturer, device trade name, keyword heading word, floating subheading word, candidate term word] 4291704<br>21 18 or 19 or 20 4291704<br>22 6 and 17 and 21 1265<br>Limited to 1990-2021 = 1228
**Web of Science**	Timespan: 1990-01-01 to 2021-09-28 (Index Date)<br><br>‘General Practitioner*’ OR ‘Primary Health Care’ OR ‘Family Physician*’ OR ‘GP*’ and ‘perinatal care’ OR ‘postpartum period’ OR postnatal care’ OR ‘maternal health’ OR ‘puerperal disorders’ OR ‘diabetes, gestational’ OR ‘pelvic organ prolapse’ OR ‘perinatal’ or ‘postpartum’ OR ‘postnatal’ and ‘perception*’ OR ‘attitude*’ OR ‘belief*’ OR ‘opinion*’ OR ‘understanding’ OR viewpoint*’ OR ‘perspective*’ OR ‘perception*’ OR ‘view*’ OR ‘experience*’<br>=223
**PubMed**	("general practitioners"[MeSH Terms] OR "primary health care"[MeSH Terms] OR "physicians, primary care"[MeSH Terms] OR "physicians, family"[MeSH Terms]OR 'general practitioner' OR 'GP') AND ("perinatal care"[MeSH Terms] OR "postpartum period"[MeSH Terms] OR "postnatal care"[MeSH Terms] OR "maternal health"[MeSH Terms] OR "puerperal disorders"[MeSH Terms] OR "diabetes, gestational"[MeSH Terms] OR "hypertension, pregnancy induced"[MeSH Terms] OR "urinary incontinence"[MeSH Terms] OR "pelvic organ prolapse"[MeSH Terms] OR ‘perinatal’ OR ‘postpartum’ OR ‘postnatal’) AND ("attitude"[MeSH Terms] OR "attitude of health personnel"[MeSH Terms] OR "perception"[MeSH Terms] OR attitude OR belief OR opinion OR understanding OR viewpoint OR perspective OR perception OR view OR experience)<br>= 1743<br><br>Limited to 1990 – 2021 = 1662
**CINAHL**	((MH "Physicians, Family") OR "general practitioner" OR "GP") AND ((MH "Postpartum Care (Saba CCC)") OR (MH "Postnatal Care") OR (MH "Postnatal Period") OR (MH "Depression, Postpartum") OR (MH "Postpartum (Omaha)") OR (MH "Puerperium") OR "("perinatal care" OR "postpartum period" OR "postnatal care" OR "maternal health" OR "puerperal disorders" OR "diabetes, gestational" OR "hypertension, pregnancy induced" OR "urinary incontinence" OR "pelvic organ prolapse" OR ‘perinatal’ OR ‘postpartum’ OR ‘postnatal’" OR (MH "Perinatal Care (Saba CCC)") OR (MH "Urinary Incontinence Care (Saba CCC)") OR (MH "Perinatal Care") OR (MH "Diabetes Mellitus, Gestational") OR (MH "Pelvic Organ Prolapse") OR (MH "Pregnancy-Induced Hypertension")) AND ("‘attitude*’ ‘belief*’ ‘opinion*’ ‘understanding*’ ‘viewpoint*’ ‘perspective*’ ‘perception*’ ‘view*’ ‘experience*’" OR (MH "Attitude of Health Personnel") OR (MH "Physician Attitudes") OR (MH "Attitude to Mental Illness") OR (MH "Attitude to Breast Feeding") OR (MH "Caregiver Attitudes") OR (MH "Attitude to Medical Treatment") OR (MH "Job Experience") OR (MH "Perception") OR (MH "Attitude"))

For peer review

Summary of study characteristics

Title	Author	Year	Setting	Recruitment	Number of GPs	Additional participants	Focus of study	Data collection from GPs	Analysis methodology	Comments
**Postnatal care following hypertensive disorders of pregnancy: a qualitative study of views and experiences of primary and secondary clinicians**	Bick et al	2019	UK	GPs invited from practices in 3 London maternity unit areas	5	Obstetricians, physician, health visitors, midwives (n=15)	Views of clinicians caring for postnatal women who had HDP	Semi-structured interviews	Thematic	
**The views of mothers and GPs about postpartum care in Australian general practice**	Brodribb et al	2013	Australia	GPs in locations serving hospitals taking part in a postnatal care study	6	Mothers (n=88)	Views about postpartum care in general practice	Interviews	Thematic	Reports on its pilot, and larger study together. Only one theme reported in full, authors contacted but no response
**GPs' and health visitors' views on the diagnosis and management of postnatal depression**	Chew-Graham et al	2008	UK	Invitation via practices who were participating in an associated RCT. Purposive by GP demographics.	19	Health visitors (n=14)	Views on role in detection and management of postnatal depression	In-depth interviews	Thematic	
**Disclosure of symptoms of postnatal depression, the perspectives of health professionals and women: a qualitative study**	Chew-Graham et al	Reports on same study as above								
**Investigating the current knowledge and needs concerning follow-up for long-term cardiovascular risks in Dutch women with a preeclampsia history: a qualitative study**	Dijkhuis et al	2020	Netherlands	Convenience, GP offices in region of hospital study base	4	Women, obstetricians, vascular medicine specialists, cardiologists (n=25)	Opinions and wishes about follow-up for women with a history of pre-ecplampsia	Semi-structured interviews	Thematic	
**Falling through the net - Black and minority ethnic women and perinatal mental healthcare: health professionals' views**	Edge	2010	UK	GPs from areas with high Black Caribbean population	5	Midwives, hospital doctors, health visitors (n=37)	Investigating views about perinatal mental healthcare for BAME women	In-depth interviews	Framework	
**Health Care Providers' Perceptions of Responsibilities and Resources to Reduce Type 2 Diabetes Risk After Gestational Diabetes Mellitus**	Hewage et al	2018	Singapore	Purposive, snowball sampling	5	OB/GYN, endocrinologists, dietitians, nurses (n=27)	Healthcare providers' perceptions of care responsibilities and resources related to reducing T2DM among women with previous GDM	In-depth interviews	Framework	
**GPs' decision-making when prescribing medicines for**	Jayawickrama et al	2010	Australia	Anonymous postal questionnaire	253	-	Factors influencing GPs' decision-making when	Survey	Thematic Networks	

**breastfeeding women: Content analysis of a survey**							prescribing for breastfeeding women			
**Falling through the gaps: perinatal mental health and general practice**	Khan	2018	UK	Convenience (large scale dissemination through virtual portals)	43	Women (n=1551)	Experiences of interactions between women with common perinatal mental health problems and GPs	Survey and semi-structured interviews	Interpretative phenomenological approaches	
**Postnatal gestational diabetes mellitus follow-up: Perspectives of Australian hospital clinicians and general practitioners**	Kilgour et al	2018	Australia	GPs who worked in collaboration with a large city hospital (where the hospital clinicians were recruited from)	16	Hospital clinicians (n=13)	Communication processes between GPs and hospital clinicians providing postnatal GDM care		Computer automated Leximancer analysis	
**Experiences of women, hospital clinicians and general practitioners with gestational diabetes mellitus follow-up: A mixed methods approach**	Kilgour et al	Reports on same study as above, but with 2 further (quant) stages. Some additional GP qual data included in this publication to be included.								
**Views of general practitioners on providing contraceptive advice and long-acting eversible contraception at the 6-week postnatal visit: a qualitative study**	Lunniss et al	2015	UK	Purposive (practice demographics), , convenience (education event), snowball	13	-	Views on contraception provision including LARCs at 6 week check	Semi-structured interviews	Thematic	
**An exploratory qualitative study exploring GPs' and psychiatrists' perceptions of post-traumatic stress disorder in postnatal women using a fictional case vignette**	Mortimer et al	2021	UK	Email invitation / poster adverts (not explicit whether purposive)	6	Psychiatrists (n=7)	GP and psychiatrist perceptions of PTSD in postnatal women	Semi-structured interviews with a case vignette	Framework	
**Irish general practitioners' view of perinatal mental health in general practice: a qualitative study**	Noonan et al	2018	Ireland	Purposive, GPs connected with GP tutors from	10	-	Caring for women with perinatal mental illness	Semi-structured interviews	Thematic	
**Improving follow-up care for women with a history of gestational diabetes: perspectives of GPs and patients**	Pennington et al	2017	Australia	Purposive (GP and practice demographics)	18	Women (n=16)	Perspectives of factors influencing engagement with diabetes prevention after a pregnancy with GDM	Semi-structured interviews	Thematic content	
**Experiences of primary care physicians managing postpartum care: a qualitative research study**	Poon et al	2021	Singapore	Purposive (demographics), networks of the researchers	29	-	Managing postpartum women	Focus Groups<br>In depth interviews	?thematic, unclear	
**Healthcare professionals' perspectives on identifying and managing perinatal anxiety: a qualitative study**	Silverwood et al	2019	UK	GPs via WM-CRN and personal contacts	10	Midwives, HVs, (n=12)	Identification and management of perinatal anxiety	Semi-structured interviews	Constant comparison	

For peer review only

**Towards an understanding of GPs' viewpoint on diagnosing postnatal depression in general practice: a small-scale realist evaluation**	Sriranjan et al	2020	UK	Convenience sample, single GP practice	7	-		Semi-structured interviews with case vignettes	Framework analysis	
**Behaviour change opportunities at mother and baby checks in primary care: a qualitative investigation of the experiences of GPs**	Talbot et al	2018	UK	Snowball sampling GP practices	18	-	Health behaviour change opportunities at the postnatal check	Semi-structured interviews - phone	Thematic analysis	
**Healthcare Professionals' Perspectives on the Cross-Sectoral Treatment Pathway for Women with Gestational Diabetes during and after Pregnancy – A Qualitative Study**	Timm et al	2021	Denmark	Purposive to cover antenatal and postnatal period, by email / phone<br>(selection not clearly stated)	2	Midwives, obstetricians, nurse, health visitors (n=15)	Management of GDM in and after pregnancy	Semi-structured interviews	Systematic Text Condensation	

For peer review only

Supplementary File 3: CASP Assessment Table

	Bick et al (2019)	*Brodribb et al (2013)*	*Chew-Graham et al (2008)*	*Dijkhuis et al (2020)*	*Edge et al (2010)*	*Hewage et al (2018)*	*Jayawickrama et al (2010)*	*Kham (2015)*	*Kilgour et al (2018*	*Lunniss et al (2015)*	*Mortimer et al (2021)*	*Noonan et al (2018)*	*Pennington et al (2017)*	*Poon et al (2021)*	*Silverwood et al (2019)*	*Sriranjan et al (2020)*	*Talbot et al (2018)*	*Timm et al (2021)*
**Was there a clear statement of the aims of the research?**	Y	Y	Y	Y	Y	Y	Y	N	Y	Y	Y	Y	Y	Y	Y	Y	Y	Y
**Is a qualitative methodology appropriate?**	Y	Y	Y	Y	Y	Y	Y	Y	Y	Y	Y	Y	Y	Y	Y	Y	Y	Y
**Was the research design appropriate to address the aims of the research?**	Y	Y	Y	Y	Y	Y	Y	Unclear	Y	Y	Y	Y	Y	Y	Y	Y	Y	Y
**Was the recruitment strategy appropriate to the aims of the research?**	Y	Y	Y	Y	Y	Y	Y	N	Y	Y	Y	Y	Y	Y	Y	Y	Y	Y
**Was the data collected in a way that addressed the research issue?**	Y	Y	Y	Y	Y	Y	N	Y	Y	Y	Y	Y	Y	Y	Y	Y	Y	Y
**Has the relationship between researcher and participants been adequately considered?**	Unclear	N	N	Y	N	N	N	N	Y	N	Y	Y	N	Y	N	Y	N	N
**Have ethical issues been taken into consideration?**	Y	Y	Y	Y	Y	Y	Y	Unclear	Y	Y	Y	Y	Y	Y	Y	Y	Y	Y
**Was the data analysis sufficiently rigorous?**	Y	Y	Y	Y	Y	Y	Y	N	Y	N	Y	Y	Y	Y	Y	Y	Y	Y
**Is there a clear statement of findings?**	Y	Y	Y	Y	Y	Y	Y	Y	Y	Y	Y	Y	Y	Y	Y	Y	Y	Y
**How valuable is the research; will the results help locally?**	Y	Y	Y	Y	Y	N	Y	Y	Y	Y	Y	Y	Y	Y	Y	Y	Y	Y

Supplementary File 4: Descriptive Themes

Descriptive Theme	Description / definition
Content and timing of postnatal checks	Any reference to content or timing of postnatal reviews, including follow-up or monitoring of pregnancy-specific pathology
Agenda setting - woman or GP	GPs' beliefs about what women want to address, or references to GPs deciding what they will cover
Baby as distraction	Baby issues taking priority, focus being baby-driven postnatally
Clinical decision making	Beliefs about diagnoses and clinical management, descriptions about how pathology is managed
Communication between other services and primary care	Communication from hospital to primary care, or other services and primary care, including notification of which patients need specific follow-up etc
Continuity of care	Specific mentions of continuity of care – either services separate from primary care (e.g. views on midwifery continuity of care) or GP views on GP continuity of care
Doctor-related factors and personal experience	Characteristics / descriptive statements about doctors that influence (positive or negative) care including personal experience
Guidelines, screening tools and prompts	Including use of guidelines, prompts or screening tools and references to their use
Knowledge	What GPs say they know, or do not know
Organisation of primary care and health visiting	GP services, including access e.g. booking appointments, arranging follow-up and descriptions about how HV is organised, including how it has changed
Referral to other services	Including accessing patient-specific advice
Role of the GP and MDT members	Descriptions of the nature of the role of the GP and other healthcare professionals e.g. HV / pharmacists / midwives
Time-related factors	Including duration of the appointment, issues that impact time pressures
Training and education	References to training received, or training wanted
Woman-related factors	GPs views on factors about women that influences care, including involvement (or not) of their partners

Mapping descriptive themes to the COM-B domains

COM-B domain	Descriptive Themes	Sources
**Psychological Capability**	Knowledge	(31, 35, 40, 41, 44, 47, 49, 50)
**Physical Capability**	None	
**Physical Opportunity**	Time-related factors<br>Screening tools or prompts<br>Organisation of primary care and health visiting<br>Referral to other services<br>Continuity of care<br>Communication between services<br>Training and education<br>Clinical guidelines, screening tools or prompts	(31-50)
**Social Opportunity**	Woman-related factors<br>Baby as distraction<br>Antenatal and intrapartum experiences<br>Agenda setting<br>GP roles<br>MDT roles	(31-42, 44-46, 48-50)
**Reflective and Automatic Motivation**	Doctor-related factors and personal experience<br>Clinical decision making	(32-34, 37, 38, 39, 43-49)

PRISMA 2020 Checklist

Section and Topic	Item #	Checklist item	Location where item is reported
**TITLE**			
Title	1	Identify the report as a systematic review.	Title p1
**ABSTRACT**			
Abstract	2	See the PRISMA 2020 for Abstracts checklist.	Abstract p2
**INTRODUCTION**			
Rationale	3	Describe the rationale for the review in the context of existing knowledge.	Background p5
Objectives	4	Provide an explicit statement of the objective(s) or question(s) the review addresses.	Aims p5
**METHODS**			
Eligibility criteria	5	Specify the inclusion and exclusion criteria for the review and how studies were grouped for the syntheses.	Methods p6-7
Information sources	6	Specify all databases, registers, websites, organisations, reference lists and other sources searched or consulted to identify studies. Specify the date when each source was last searched or consulted.	Information sources p8
Search strategy	7	Present the full search strategies for all databases, registers and websites, including any filters and limits used.	Supp file 1
Selection process	8	Specify the methods used to decide whether a study met the inclusion criteria of the review, including how many reviewers screened each record and each report retrieved, whether they worked independently, and if applicable, details of automation tools used in the process.	Screening and data extraction p8
Data collection process	9	Specify the methods used to collect data from reports, including how many reviewers collected data from each report, whether they worked independently, any processes for obtaining or confirming data from study investigators, and if applicable, details of automation tools used in the process.	Screening and data extraction p8
Data items	10a	List and define all outcomes for which data were sought. Specify whether all results that were compatible with each outcome domain in each study were sought (e.g. for all measures, time points, analyses), and if not, the methods used to decide which results to collect.	Data synthesis p8-9
	10b	List and define all other variables for which data were sought (e.g. participant and intervention characteristics, funding sources). Describe any assumptions made about any missing or unclear information.	Data synthesis p8-9
Study risk of bias assessment	11	Specify the methods used to assess risk of bias in the included studies, including details of the tool(s) used, how many reviewers assessed each study and whether they worked independently, and if applicable, details of automation tools used in the process.	Quality appraisal p9
Effect measures	12	Specify for each outcome the effect measure(s) (e.g. risk ratio, mean difference) used in the synthesis or presentation of results.	n/a
Synthesis methods	13a	Describe the processes used to decide which studies were eligible for each synthesis (e.g. tabulating the study intervention characteristics and comparing against the planned groups for each synthesis (item #5)).	n/a
	13b	Describe any methods required to prepare the data for presentation or synthesis, such as handling of missing summary statistics, or data conversions.	Data synthesis p8-9
	13c	Describe any methods used to tabulate or visually display results of individual studies and syntheses.	Data synthesis p8-9
	13d	Describe any methods used to synthesize results and provide a rationale for the choice(s). If meta-analysis was performed, describe the model(s), method(s) to identify the presence and extent of statistical heterogeneity, and software package(s) used.	Data synthesis p8-9
	13e	Describe any methods used to explore possible causes of heterogeneity among study results (e.g. subgroup analysis, meta-regression).	Data synthesis p8-9
	13f	Describe any sensitivity analyses conducted to assess robustness of the synthesized results.	n/a
Reporting bias	14	Describe any methods used to assess risk of bias due to missing results in a synthesis (arising from reporting biases).	Data synthesis

For peer review only - http://bmjopen.bmj.com/site/about/guidelines.xhtml

PRISMA 2020 Checklist

Section and Topic	Item #	Checklist item	Location where item is reported
assessment			p8-9
Certainty assessment	15	Describe any methods used to assess certainty (or confidence) in the body of evidence for an outcome.	n/a
**RESULTS**			
Study selection	16a	Describe the results of the search and selection process, from the number of records identified in the search to the number of studies included in the review, ideally using a flow diagram.	Results p9, fig1
	16b	Cite studies that might appear to meet the inclusion criteria, but which were excluded, and explain why they were excluded.	Included studies p9
Study characteristics	17	Cite each included study and present its characteristics.	Supp file2
Risk of bias in studies	18	Present assessments of risk of bias for each included study.	Supp file3
Results of individual studies	19	For all outcomes, present, for each study: (a) summary statistics for each group (where appropriate) and (b) an effect estimate and its precision (e.g. confidence/credible interval), ideally using structured tables or plots.	n/a
Results of syntheses	20a	For each synthesis, briefly summarise the characteristics and risk of bias among contributing studies.	Results/Themes p9-16
	20b	Present results of all statistical syntheses conducted. If meta-analysis was done, present for each the summary estimate and its precision (e.g. confidence/credible interval) and measures of statistical heterogeneity. If comparing groups, describe the direction of the effect.	n/a
	20c	Present results of all investigations of possible causes of heterogeneity among study results.	n/a
	20d	Present results of all sensitivity analyses conducted to assess the robustness of the synthesized results.	n/a
Reporting biases	21	Present assessments of risk of bias due to missing results (arising from reporting biases) for each synthesis assessed.	n/a
Certainty of evidence	22	Present assessments of certainty (or confidence) in the body of evidence for each outcome assessed.	n/a
**DISCUSSION**			
Discussion	23a	Provide a general interpretation of the results in the context of other evidence.	Discussion p16-17
	23b	Discuss any limitations of the evidence included in the review.	Discussion p16-17
	23c	Discuss any limitations of the review processes used.	Discussion p16-17
	23d	Discuss implications of the results for practice, policy, and future research.	Discussion p16-17
**OTH ER INFORMATION**			
Registration and protocol	24a	Provide registration information for the review, including register name and registration number, or state that the review was not registered.	Cover page p1
	24b	Indicate where the review protocol can be accessed, or state that a protocol was not prepared.	PROSPERO
	24c	Describe and explain any amendments to information provided at registration or in the protocol.	PROSPERO

For peer review only

PRISMA 2020 Checklist

Section and Topic	Item #	Checklist item	Location where item is reported
Support	25	Describe sources of financial or non-financial support for the review, and the role of the funders or sponsors in the review.	Cover page p1
Competing interests	26	Declare any competing interests of review authors.	p18
Availability of data, code and other materials	27	Report which of the following are publicly available and where they can be found: template data collection forms; data extracted from included studies; data used for all analyses; analytic code; any other materials used in the review.	Supplementary files

From: Page MJ, McKenzie JE, Bossuyt PM, Boutron I, Hoffmann TC, Mulrow CD, et al. The PRISMA 2020 statement: an updated guideline for reporting systematic reviews. BMJ 2021;372:n71. doi: 10.1136/bmj.n71

For more information, visit: http://www.prisma-statement.org/

For peer review only
